# Mutation and Selection in Bacteria: Modelling and Calibration

**DOI:** 10.1007/s11538-018-0529-9

**Published:** 2018-11-14

**Authors:** C. D. Bayliss, C. Fallaize, R. Howitt, M. V. Tretyakov

**Affiliations:** 1 Department of Genetics, University of Leicester, Leicester, LE1 7RH UK; 2 School of Mathematical Sciences, University of Nottingham, University Park, Nottingham, NG7 2RD UK

**Keywords:** Stochastic modelling, Population genetics, Phase variable genes, Approximate Bayesian computation, 92D25, 62F15, 60J10

## Abstract

Temporal evolution of a clonal bacterial population is modelled taking into account reversible mutation and selection mechanisms. For the mutation model, an efficient algorithm is proposed to verify whether experimental data can be explained by this model. The selection–mutation model has unobservable fitness parameters, and, to estimate them, we use an Approximate Bayesian Computation algorithm. The algorithms are illustrated using in vitro data for phase variable genes of *Campylobacter jejuni*.

## Introduction

The objective of this paper is to propose stochastic models for bacterial population genetics together with their calibration. In other words, our aim is not only to construct models but also to suggest algorithms which can answer the question as to whether experimental data can be explained by a model or not. An answer to this question is the key for establishing which mechanisms are dominant in evolution of bacteria. The models are deliberately relatively simple though they capture two important mechanisms of bacterial population genetics: mutation and selection. Simplicity of the models allows their fast calibration, and it is also consistent with the fact that in experiments sample sizes are usually relatively small.

The models are derived, calibrated and tested within the context of phase variable (PV) genes, which occur in many bacterial pathogens and commensals (Bayliss 2009; Bayliss et al. 2012). Phase variation has three properties: (i) an on/off or high/low switch in gene expression; (ii) high switching rates; and (iii) reversible switching between expression states. Two major mechanisms of phase variation involve hypermutable simple sequence repeats (SSR) and high-frequency site-specific recombinatorial changes in DNA topology (Bayliss 2009; Bayliss et al. 2012; Wisniewski-Dyé and Vial 2008; van der Woude and Bäumler 2004; Moxon et al. 1994). We note that in contrast to phase variation, non-PV mutations have lower rates and extremely rare reverse mutations, while PV genes have high mutation rates (e.g. in the case of *Campylobacter jejuni* they are estimated to fall between $4\times 10^{-4}$ and $4 \times 10^{-3}$). PV genes can lead to changes in the expression of outer membrane proteins or structural epitopes of large surface molecules whose functions modulate multiple interactions between bacteria and hosts including adhesion, immune evasion and iron acquisition. Consequently, phase variation can influence host adaptation and virulence. Models accompanied by efficient data assimilation procedures are an important tool for understanding adaptation of bacteria to new environments and ultimately for determining how some bacteria cause disease.

SSR-mediated phase variation is considered herein as this is the specific mechanism occurring in genes of *C. jejuni* which we will use in our illustrative examples. SSR, otherwise known as microsatellites, consist of tandem arrangements of multiple copies of an identical sequence (i.e. the repeat). In *C. jejuni*, the majority of these SSR consist of non-triplet repeats, polyG or polyC, present within the reading frame. Between 18 and 39 PV genes are present in each *C. jejuni* strain (Aidley et al. 2018). SSR tracts are hypermutable due to a high error rate occurring during DNA replication. Slipped strand mispairing, the proposed mechanism (Levinson and Gutman 1987), alters gene expression through parent and daughter strand misalignment during replication, which results in deletion or addition of one repeat unit in the newly-synthesized strand. Changes in repeat number of a non-triplet repeat present within a reading frame alter the coding sequence of the codon triplets producing switches in gene expression, and hence the switches in phenotypes referred to as phase variation.

Other modelling approaches to bacterial population genetics can be found in, e.g. Alonso et al. (2014), Saunders et al. (2003) and Moxon and Kussell (2017) (see also references therein). These models have explored the interplay between selection, mutation and population structure for multiple interacting genes with low or high mutation rates and varying levels of selection (Alonso et al. (2014); Gerrish et al. (2013); Palmer and Lipsitch (2006); Wolf et al. (2005); Barrick and Lenski (2013); O’Brien et al. (2013); Raynes and Sniegowski (2014)). A subset of these models have explicitly focused on hypermutability, where reversion is a defining and important phenomenon. These models have indicated that evolution of hypermutability is driven by the strength and period of selection for each expression state but is also influenced by the frequency of imposition of population bottlenecks (Saunders et al. (2003); Moxon and Kussell (2017); Palmer et al. (2013); Acar et al. (2008)). The majority of these models have considered single-gene phenomena and have not provided approaches or adjustable, portable models for application to actual experimental observations. An exception is the use of a model of non-selective bottlenecks of PV genes Aidley et al. (2017) that was utilized to predict the bottleneck size in observed bacterial populations Wanford et al. (2018). The aim herein is to develop models that could be used to examine experimentally observed populations and determine whether mutation rate alone or mutation rate and selection for changes in expression of one or more loci were driving changes in bacterial population structure. Our main focus here is on host adaptation of a clonal population of hypermutable bacteria, for which we propose a mutation–selection model. The model describes collective behaviour of interactive PV genes and is accompanied by an effective data assimilation procedure.

The rest of the paper is organized as follows. In Sect. 2, we first recall and revise the mutation model from Bayliss et al. (2012), which is a stochastic discrete-time discrete-space model describing the mutation mechanism only. It is derived under the assumptions of infinite (very large) size of the population maintained during the whole time period of interest, time is measured in generations, and all phasotypes have the same survival rate (fitness). Then, we introduce a new model (mutation–selection model) which takes into account both mutation and selection mechanisms. It generalizes the mutation model by allowing phasotypes to have different fitness levels. We also discuss properties, including long-time behaviour, of both models. Then, we turn our attention to calibration of the models. In Sect. 3, we propose a very efficient algorithm to test whether experimental data can be explained by the mutation model from Sect. 2 and we illustrate the algorithm by applying it to in vitro data for three PV genes of *C. jejuni*. In Sect. 4, we describe general methodology for estimating fitness parameters (as well as other quantities) in the mutation–selection model using Approximate Bayesian Computation (ABC), as well as an algorithm for detecting lack of independence between fitness parameters of different genes. In Sect. 5, we illustrate the methodology with applications to synthetic and real data from experiments involving the bacteria *C. jejuni.* We conclude with a discussion.

## Models

Assume that a population of bacteria is sufficiently large (for theoretical purposes “near” infinite). As we will see later in this section, this assumption is used in constructing the models to average over branching trees occurring during population evolution in order to have deterministic dynamics of phasotype distributions. Hence, the required population size depends on the number of genes considered (the more genes, the richer the state space of the models and a larger population size is required) and on transition (mutation) rates. (Rare events need to be “recorded” in the population.) This simplifying assumption allows us to have tractable models which can be efficiently calibrated as we show in Sects. 3, 4 and 5. Using the models, we can examine large bacterial populations, say of size 10,000 or more, which is biologically relevant when the population is far from extinction (this situation is relevant to weak selection but may not be applicable to very strong selective pressures that cause high mortality rates and significant reductions in population size) and far from so-called bottlenecks as may occur due to strong selection or during transmission of bacterial populations between hosts or other environmental niches. The latter deserves a separate modelling and study (see, e.g. Aidley et al. 2017; Moxon and Kussell 2017).

In modelling, we neglect the continuous-time effect (see, e.g. Crow and Kimura 1970) and measure time as numbers of generations. The number of generations between two time points is evaluated as the time between the points multiplied by an average division rate. The rate can be estimated in experiments by measuring how much time is required for a population to double in the absence of selection. This simplifying assumption neglects effects related to random time of bacterial division. To compensate the use of average division rate, in calibration (Sects. 3, 4, 5) we assign to each time point a range of possible numbers of generations occurred since the previous observation.

We describe each bacterium via a status of its $\ell$ PV genes each of which can be either in the state OFF or ON. The OFF and ON states are coded as 0 and 1, respectively. Hence, we can represent the phasotype of each bacterium as a random vector

1 $$\begin{aligned} \xi =\left( \xi _{1},\ldots ,\xi _{\ell }\right) , \end{aligned}$$

where $\xi _{i}$ can take only two values, 0 or 1. The random vector $\xi$ has $2^{\ell }$ possible values from the state space

2 $$\begin{aligned} \varOmega =\left\{ A_{i}=\left( a_{i1},\ldots ,a_{i\ell }\right) \text { with } a_{ij}=0,1\right\} , \end{aligned}$$

where we label each element $$A_{i}$$ of $$\varOmega $$ by a number *i* from 1 to $$2^{\ell }$$ in the increasing order of the corresponding binary numbers: $$ A_{1}=\left( 0,\ldots ,0\right) ,$$$$A_{2}=\left( 0,\ldots ,0,1\right) ,$$$$ \ldots ,$$$$A_{2^{\ell }}=\left( 1,\ldots ,1\right) .$$

### Remark 2.1

We assume that $\xi _{i}$ can take only two values 0 and 1 since this work is mainly motivated by PV genes as explained in the Introduction. To study more detailed genome evolution of bacteria (e.g. repeat numbers instead of phasotypes), the models presented in this section can be easily generalized to the case when the random variables $\xi _{i}$, $i=1,\ldots ,\ell ,$ can take more than two values without need of additional ideas (see, e.g. Hardwick et al. 2009, where a mutation model analogous to the one presented in Sect. 2.1 but with multiple values of $\xi _{i}$ was used). However, for clarity of the exposition we restrict ourselves to the binary case here.

In Sect. 2.1, we derive a discrete-time discrete-space stochastic model for evolution of phasotypes after a fixed number of generations *n*, taking into account only the mutation mechanism of genes. (This shall be referred to herein as the mutation model.) This model was proposed in Bayliss et al. (2012) (see also Hardwick et al. 2009); here, we provide more details which are needed for clarity of exposition. In Sect. 2.2, a discrete-time discrete-space stochastic model is considered for the binary switching in bacteria which takes into account fitness of genes in addition to mutation. (This shall be referred to herein as the mutation–selection model.) In Sect. 2.3, it will be shown when unique stationary distributions exist for both models.

### Genetic Drift Modelling

Consider a parent bacterium at time $n=0$ whose phasotype is $x\in \varOmega .$ At (discrete) time $n=1$ (i.e. after the first cell division) the parent bacterium produces two offspring: $\xi (1;1;x)\in \varOmega$ and $\xi (1;2;x)\in \varOmega ,$ which are assumed to be conditionally (conditioned on the initial state *x*) independent random vectors. This conditional independence assumption is natural for a mutation process and has been utilized in similar models (Hardwick et al. 2009; Palmer and Lipsitch 2006; Bayliss et al. 2012). We introduce the transitional probabilities

3 $$\begin{aligned} p_{ij}=\mathbb {P}\left( \xi (1;1;x)=A_{j}|x=A_{i}\right) =\mathbb {P}\left( \xi (1;2;x)=A_{j}|x=A_{i}\right) \end{aligned}$$

from which we form the $2^{\ell }\times 2^{\ell }$ matrix of transitional probabilities $T=\left\{ p_{ij}\right\} .$ It is natural to assume that

4 $$\begin{aligned} p_{ij}>0\quad \text { for all }\quad i,j. \end{aligned}$$

Let us make the following assumption which can be interpreted as stationarity of mutation rates.

#### Assumption 2.1

Assume that the matrix of transitional probabilities *T* does not change with time.

Now we continue with the dynamics so that at time $$n=2$$ the bacteria $$\xi (1;1;x)$$ and $$\xi (1;2;x)$$ produce their four offspring, then at time $$n=3$$ we get eight bacteria, and so on. (For the time being, we assume that no bacteria are dying before producing offspring.) As a result, we obtain a binary branching tree. Denote by $$Z_{k}(n|x)$$ the number of bacteria of type $$ A_{k}$$ in the population after *n* divisions starting from the bacterium of type *x* at time zero. This number is clearly random as it depends on a realization $$\omega $$ of the branching tree and its more detailed notation is $$Z_{k}(n|x)(\omega ).$$ The collection

$$\begin{aligned} Z(n|x)(\omega )=\left\{ Z_{k}(n|x)(\omega ),\ k=1,\ldots ,2^{\ell }\right\} \end{aligned}$$

describes a population living on the set $\varOmega$ and the total amount of bacteria after *n* divisions is $2^{n}:$

$$\begin{aligned} \sum _{k=1}^{2^{\ell }}Z_{k}(n|x)(\omega )=2^{n}. \end{aligned}$$

Let us randomly (i.e. independently) draw a member, i.e. a bacterium with a PV state, from this population and ask the question: what is the probability of the PV state being $A_{k}$? Obviously, for a fixed $\omega$ (i.e. for a particular realization of the branching tree), the probability to pick a bacterium of the type $A_{k}$ is equal to

5 $$\begin{aligned} \rho _{k}(n|x)(\omega )=\frac{1}{2^{n}}Z_{k}(n|x)(\omega ). \end{aligned}$$

This is a random distribution which is analogous to random measures appearing in Wright–Fisher-type models (Crow and Kimura 1970). Since we are interested in the situation when a population of bacteria is of “near” infinite size, we will characterize the bacteria population at every time by an average of the distribution $\rho _{k}(n|x)(\omega ),$ where averaging is done over all possible realizations of the branching trees.

If we put together all possible realizations of the branching trees with the corresponding random unnormalized distributions $$Z(n|x)(\omega _{1}),$$$$ Z(n|x)(\omega _{2}), \ldots $$, then the proportion of bacteria of the type $$ A_{k}$$ in this total population of bacteria is equal to

6 $$\begin{aligned} \pi ^{k}(n|x)=\sum _{j=1}^{2^{n}}\frac{j}{2^{n}}\mathbb {P}\left( Z_{k}(n|x)=j\right) =\frac{1}{2^{n}}\mathbb {E}Z_{k}(n|x)=\mathbb {E} \rho _{k}(n|x). \end{aligned}$$

The meaning of the average $\pi ^{k}(n|x)$ is as follows. If we consider all possible binary trees (created via division of bacteria as discussed earlier) which started from a bacterium in state *x*, and we look at the resulting total bacteria population after *n* divisions, then the proportion of bacteria with PV type $A_{k}$ in this total population is given by the average $\pi ^{k}(n|x).$ We note that $\pi (n|x):=(\pi ^{1}(n|x),\ldots ,\pi ^{2^{\ell }}(n|x))$ is a distribution defined on the set $\varOmega .$ The distribution $\pi (n|x)$ is well suited for modelling in the typical experimental setting when studying evolution of bacteria. Indeed, in both in vitro and in vivo experiments with bacteria we usually cannot observe evolution of a particular bacterium (i.e. a particular binary tree). Instead, a sample is collected from a large bacteria population at particular time points and data (the motivation for this paper is PV data) are extracted for this sample. So, in experiments one typically observes a sample distribution $_{i}\hat{\pi }$ at a time point *i* and, by tending the sample size to infinity, $_{i}\hat{\pi }$ converges (under the standard assumptions for the law of large numbers, and it is natural to assume that for the considered application these assumptions hold) to an average distribution $_{i}\pi ,$ which we model using $\pi (n|x).$ We will link the models considered in this Section with experimental data in Sects. 3 and 4.

Now let us show that time evolution of the measures $\pi (n|x)$ resembles evolution of the distribution for a (linear) Markov chain. Using the previously stated assumption of conditional independence between the states of daughters of the parent bacterium, and the transitional probabilities $p_{ij}$ from (3), we get

$$\begin{aligned} \mathbb {E}Z_{k}(1|x=A_{i})=0\times (1-p_{ik})^{2}+2p_{ik}(1-p_{ik})+2p_{ik}^{2}=2p_{ik}, \end{aligned}$$

then

$$\begin{aligned} \pi ^{k}(1|x=A_{i})=p_{ik} \end{aligned}$$

and

$$\begin{aligned} \pi (1|x=A_{i})=\pi (0)T, \end{aligned}$$

where $\pi (0)$ is a vector in which all components are equal to zero except the *i*th component being equal to 1 (recall that at this stage we assume that at time zero we had just a single bacterium in the state $A_{i}$). Analogously, we obtain

$$\begin{aligned} \pi ^{k}(2|x=A_{i})=\sum _{j=1}^{2^{\ell }}p_{ij}p_{jk} \end{aligned}$$

and

$$\begin{aligned} \pi ^{k}(n|x=A_{i})=\sum _{j=1}^{2^{\ell }}\pi ^{j}(n-1|x=A_{i})p_{jk}. \end{aligned}$$

Hence,

7 $$\begin{aligned} \pi (n|x=A_{i})=\pi (0)T^{n}. \end{aligned}$$

We see that the time evolution of the population distribution resembles evolution of a distribution of states of a linear Markov chain. But we emphasize that the underlying model is not a Markov chain, since it is obtained by averaging over branching trees rather than modelling an individual by a Markov chain on the state space. The resemblance is in the evolution dynamics (7) of the distribution resulting from our model, which are the same as the dynamics of a distribution of a Markov chain on the same state space. As we will see in Sect. 2.3, this resemblance is useful for studying the time limit of the evolution of $\pi (n).$

Three generalizations of model (7) are straightforward. First, instead of starting with a single bacterium at time $n=0$, we can start with a bacteria population having an initial distribution $\pi (0)$ of PV states and, consequently, we can write the *mutation model* as

8 $$\begin{aligned} \pi (n;\pi (0))=\pi (0)T^{n}. \end{aligned}$$

In the language of branching trees used above, this generalization can be interpreted in the following way. The initial state (the seeding node) $$x\in \varOmega $$ of branching trees is now a random variable with the distribution $$ \pi (0),$$ i.e. the initial state for each of the trees is randomly drawn from $$\pi (0).$$ The average distribution $$\pi (n;\pi (0))$$ in (8) is obtained by averaging not only over all possible branching trees starting from a particular state *x* as in the case of (7) but also by averaging over all possible initial states distributed according to $$\pi (0).$$ Second, so far we have been assuming that all offspring survive, and hence the population grows exponentially. However, model (8) remains valid when the number of bacteria of each type $$ A_{k}$$ at time *n* is proportional to $$\pi ^{_{k}}(n;\pi (0))$$ under the condition that the population size remains sufficiently large. The biological meaning of this assumption is that all phasotypes have the same survival rate, or in other words, the same fitness. The case when various phasotypes have different fitness is considered in Sect. 2.2. We note that since we assume the population size to remain large, it implies that the mortality rate is relatively low so that either the population size is not decreasing or decreasing relatively slowly during the time period of interest. Third, Assumption 2.1 can be relaxed to allow time dependence of the transition probabilities *T*, but the standard point of view is that mutation rates for bacteria do not change with time, and hence we do not consider this generalization here.

For clarity of the exposition, let us summarize what is meant by the mutation model in this paper, highlighting all the assumptions made during its derivation.

**Mutation Model***Under the assumptions*

- *infinite (very large) size of the population maintained during the whole time period of interest;*
- *time is measured in generations;*
- *each gene can be either in state *0 * or * 1 *(i.e. OFF or ON);*
- *all phasotypes have the same survival rate (fitness);*
- *the matrix Tof transitional probabilities does not change with time* (*Assumption* 2.1);

*we call dynamics * (7) * of the distribution *$\pi (n;\pi (0))$**the mutation model**.

It is commonly viewed that mutation of individual genes happens independently of each other, which in our phase variation context means that on/off switches of individual genes due to the mutation mechanism are independent of each other. Consequently, we can write the transition probabilities as

9 $$\begin{aligned} p_{ij}=\prod \limits _{m=1}^{\ell }p_{m}^{\alpha (i,j;m;0,1)}(1-p_{m})^{\alpha (i,j;m;0,0)}q_{m}^{\alpha (i,j;m;1,0)}(1-q_{m})^{\alpha (i,j;m;1,1)}, \end{aligned}$$

where

10 $$\begin{aligned} p_{i}= & {} \mathbb {P}\left\{ \xi _{i}(1;r;x)=1|x_{i}=0\right\} \text {, }q_{i}= \mathbb {P}\left\{ \xi _{i}(1;r;x)=0|x_{i}=1\right\} , \nonumber \\ r= & {} 1,2,\ \ i=1,\ldots ,2^{\ell }, \end{aligned}$$

and $\alpha (i,j;m;l,k)=1$ if $A_{i}$ in (2) has the $m\hbox {th}$ component equal to *l* and $A_{j}$ in (2) has the $m\hbox {th}$ component equal to *k*,  otherwise $\alpha (i,j;m;l,k)=0.$ Under the independence assumption, the matrix of transitional probabilities *T* can therefore be written using Kronecker tensor products as

11 $$\begin{aligned} T=T_{1}\otimes \cdots \otimes T_{\ell }, \end{aligned}$$

where $T_{i}$ is a $2\times 2$-matrix of transition probabilities for the *i* th gene

12 $$\begin{aligned} T_{i}=\left[ \begin{array}{cc} 1-p_{i} &{} p_{i} \\ q_{i} &{} 1-q_{i} \end{array} \right] . \end{aligned}$$

Let us formalize the independence assumption and also require that all the elements of the matrix *T* are positive.

#### Assumption 2.2

Assume that the matrix of transitional probabilities *T* for $\ell$ genes has form (11) and

13 $$\begin{aligned} 0<p_{i}<1\ \textit{and }0<q_{i}<1,\ i=1,\ldots ,2^{\ell }. \end{aligned}$$

Note that under Assumption 2.2, we have

14 $$\begin{aligned} T^{n}=T_{1}^{n}\otimes \cdots \otimes T_{\ell }^{n}. \end{aligned}$$

Further, one can show that model (8) under Assumption 2.2 implies that the evolution of individual genes is given by

15 $$\begin{aligned} \pi _{l}(n,\pi _{l}(0))=\pi _{l}(0)T_{l}^{n}\ ,\ l=1,\ldots ,\ell , \end{aligned}$$

where $\pi _{l}=(\pi _{l}^{1},\pi _{l}^{2})$ are marginal distributions for the *l*th gene, i.e.

16 $$\begin{aligned} \pi _{l}^{1}=\sum _{i=1}^{2^{\ell }}\alpha (i;l,0)\pi ^{i},\ \ \pi _{l}^{2}=\sum _{i=1}^{2^{\ell }}\alpha (i;l,1)\pi ^{i}, \end{aligned}$$

with $\alpha (j;l,k)=1$ if $A_{j}$ in (2) has the *l*th component equal to *k*,  otherwise $\alpha (j;l,k)=0.$ We see from (15) that in the case of the mutation model we can study behaviour of individual genes independently. In particular, we can verify whether data can be explained by the mutation model (8) by looking at each gene individually using (15). This will be exploited in Sect.  3.

### Mutation–Selection Model

In the previous section, we constructed a mutation model in which it was assumed that all phasotypes have the same fitness. In this section, we will generalize model (7) to include selection. By selection we mean that bacteria with some phasotypes grow faster than bacteria with other phasotypes. To take into account both mutation and selection mechanisms in modelling, we exploit the idea of splitting the dynamics. Without selection, we model mutation using (8) introduced in the previous section. Assuming there is no mutation, we can model selection via re-weighting a distribution of the population at each discrete time. Using the idea of splitting, at each discrete-time moment we first take into account the mutation mechanism using one step of (8) and then we re-weight the resulting population distribution to model the selection mechanism. We now derive the mutation–selection model.

Let us measure time in units of a typical division time for the slowest growing phasotype $A_{i}$ of the bacteria. We assume that the number of bacteria with this phasotype changes per time step by a factor

$$\begin{aligned} 0<\beta \le 2. \end{aligned}$$

Note that if all offspring survive then $$\beta =2.$$ Bacteria with the other phasotypes $$A_{j},$$$$j\ne i,$$ can be fitter and hence can grow faster per division step of the slowest growing phasotype $$A_{i}$$, with a factor of $$ \gamma _{j}\beta $$, where $$\gamma _{j}\ge 1.$$ We note that if $$\gamma _{j}=1$$ then the phasotype $$A_{j}$$ has the same growth speed as the slowest phasotype $$A_{i},$$ for which obviously $$\gamma _{i}=1.$$ The parameters $$ \gamma _{j}$$ are interpreted biologically as relative fitness of phasotypes $$ A_{j}$$ with respect to the slowest growing phasotype $$A_{i}.$$

Suppose that the total bacteria population at time *n* has a sufficiently large size *N* and its distribution is $\tilde{\pi }(n)$ “before selection”. Then, we have the following amount of bacteria per type “before selection” :

$$\begin{aligned} N_{j}=\tilde{\pi }^{j}(n)N. \end{aligned}$$

Here, $\tilde{\pi }(n)$ is obtained from population distribution $\pi _{\text { sel}}(n-1)$ at time $n-1$ according to one step of (8):

17 $$\begin{aligned} \tilde{\pi }(n)=\pi _{\text {sel}}(n-1)T. \end{aligned}$$

Selection can be modelled by re-weighting the distribution according to the relative fitness coefficients $\gamma _{j}.$ Hence, “after selection”, we have the amount of bacteria per type

$$\begin{aligned} N_{j}^{\text {sel}}=\gamma _{j}\beta \tilde{\pi }^{j}(n)N \end{aligned}$$

and the new total size of the population

$$\begin{aligned} N^{\text {sel}}=N\beta \sum _{j=1}^{2^{\ell }}\gamma ^{j}\tilde{\pi }^{j}(n). \end{aligned}$$

Therefore, the new distribution which takes selection into account is computed as

18 $$\begin{aligned} \pi _{\text {sel}}^{j}(n)=\frac{\gamma ^{j}\tilde{\pi }^{j}(n)}{ \sum _{j=1}^{2^{\ell }}\gamma ^{j}\tilde{\pi }^{j}(n)}. \end{aligned}$$

Note that our requirement for the population to be of a sufficiently large size ensures that all $N_{j}^{\text {sel}}$ remain sufficiently large so that the averaging used in Sect. 2.1 to derive the mutation model (8) can be performed. Thus, the *mutation–selection* model takes the form

19 $$\begin{aligned} \pi _{\text {sel}}(n)=\pi _{\text {sel}}(n,\pi (0),\gamma )=\frac{\pi _{\text { sel}}(n-1)TI_{\gamma }}{\gamma \cdot \pi _{\text {sel}}(n-1)T}, \end{aligned}$$

where $\gamma =(\gamma ^{1},\ldots ,\gamma ^{2^{\ell }})$ and $I_{\gamma }=$ diag$(\gamma ).$ In future, we will also use a more detailed notation

20 $$\begin{aligned} \pi _{\text {sel}}(n)=\pi _{\text {sel}}(n,p,q,\pi (0),\gamma ), \end{aligned}$$

where $$p=(p_{1},\ldots ,p_{\ell })$$ and $$q=(q_{1},\ldots ,q_{\ell }).$$

For clarity of the exposition, let us summarize what is meant by the mutation–selection model in this paper, highlighting all the assumptions made during its derivation.

**Mutation–selection Model***Under the assumptions*

- *infinite (very large) size of the population maintained during the whole time period of interest;*
- *time is measured in generations;*
- *each gene can be either in state *0 * or *1;
- *the matrix T of transitional probabilities does not change with time* (*Assumption* 2.1);
- *the vector *$\gamma$* of fitness coefficients does not change over time and all*$\gamma ^i \ge 1$;

*we call nonlinear dynamics *(19) * of the distribution *$\pi _{\text {sel}}(n)$**the mutation–selection model**.

We remark that model (19) degenerates to the mutation model (8) when all $\gamma ^{j}=1.$

Model (19) resembles a nonlinear Markov chain (Kolokoltsov 2010). Indeed, we can rewrite (19) as

21 $$\begin{aligned} \pi _{\text {sel}}(n)=\pi _{\text {sel}}(n-1)\mathbb {T}\left( \pi _{\text {sel} }(n-1)\right) , \end{aligned}$$

where $\mathbb {T}$ is a stochastic matrix which gives nonlinear transitional probabilities. We can choose $\mathbb {T}$ as

22 $$\begin{aligned} \mathbb {T}^{ij}\left( \pi _{\text {sel}}(n-1)\right) =\frac{\gamma ^j \sum _{k=1}^{\ell } \pi ^k _{\text {sel}}(n-1) T^{kj}}{\gamma \cdot \pi _{\text {sel}}(n-1)T}. \end{aligned}$$

As we will see in Sect. 2.3, this resemblance is useful for studying the time limit of the evolution of $\pi _{\text {sel}}(n)$. The stochastic representation (21) for the continuous mapping

23 $$\begin{aligned} \varPhi (\pi )=(\varPhi ^1(\pi ),\ldots ,\varPhi ^{2^\ell }(\pi )):=\frac{\pi T I_{\gamma }}{\gamma \cdot \pi T} \end{aligned}$$

is not unique unless the condition that $\mathbb {T}^{ij}=\varPhi ^j$ is imposed under which representation (21), (22), is unique (Kolokoltsov 2010, Ch. 1).

In model (19), it was assumed that the vector of fitness coefficients $\gamma$ does not change over time. But it is straightforward to generalize model (19) to the case of time-dependent fitness parameters $\gamma (n)$ by just replacing $\gamma$ in the right-hand side of (19) by $\gamma (n).$ This generalization is important for modelling adaptation of bacteria to different environments, which will be illustrated in Sect. 5.4.

In model (19), we assigned fitness coefficients $$\gamma ^{j}$$ per phasotypes $$A_{j}.$$ In our biological context, Fisher’s assumption about selection (Fisher 1930; Waxman and Welch 2005) implies that each gene contributes independently to fitness of a phasotype. In other words, if $$\gamma _{l}=(\gamma _{l}^{1},\gamma _{l}^{2}),$$$$\gamma _{l}^{i}\ge 1,$$$$\min \gamma _{l}^{i}=1,$$ describes fitness of the OFF (the first component $$ \gamma _{l}^{1})$$ and ON states (the second component $$\gamma _{l}^{2})$$ of a gene *l* then the fitness coefficient $$\gamma ^{j}$$ for the phasotype $$ A_{j}$$ can be written as the product

24 $$\begin{aligned} \gamma ^{j}=\prod \limits _{l=1}^{\ell }[\gamma _{l}^{1}]^{\alpha (j;l;0)}[\gamma _{l}^{2}]^{\alpha (j;l;1)}, \end{aligned}$$

where $\alpha (j;l,k)$ was introduced after (16) in the previous section, and we can rewrite (24) in the tensor form

25 $$\begin{aligned} \gamma =\gamma _{1}\otimes \cdots \otimes \gamma _{\ell }. \end{aligned}$$

Let us formally state this assumption.

#### Assumption 2.3

Assume that the fitness vector $\gamma$ can be expressed as the tensor product (25).

Note that under Assumption 2.3 the diagonal matrix $I_{\gamma }$ can also be written as the tensor product

26 $$\begin{aligned} I_{\gamma }=I_{\gamma _{1}}\otimes \cdots \otimes I_{\gamma _{\ell }}, \end{aligned}$$

where $$I_{\gamma _{i}}=$$ diag$$(\gamma _{i}).$$

Model (19) with the choice of fitness vector in the form of (25) is clearly a particular case of model (19) in which fitness coefficients are assigned to each phasotype individually. Let us denote this particular case by (19), (25). In comparison with (19), (25), the general model (19) can describe bacterial population evolution when individual gene dynamics are dependent on each other. This feature of the selection model is important. For instance, in the recent studies (Woodacre et al. 2019; Lango-Scholey et al. 2019; Howitt 2018) of PV genes of *C. jejuni*, evidence of small networks of genes exhibiting dependent evolutionary behaviour was found. Fisher’s assumption, and hence model (19), (25) with independent contribution of genes to fitness of phasotypes, is open to criticism (see Waxman and Welch 2005 and references therein). In Sect. 4, we describe an algorithm (Algorithm 4.2) which allows us to test whether the data can be explained by the simplified model (19), (25) or whether assumption (25) is not plausible. At the same time, model (19), (25) is simpler than the general model (19). Model (19) has $2^{\ell }-1$ (one of the fitness coefficients in (19) is equal to 1 due to normalization used in the model’s derivation) independent fitness parameters, while (19), (25) has only $\ell$ independent fitness parameters. In practice, the benefit of reducing the number of parameters by preferring (19), (25) over (19) must be weighed against the lack of versatility that arises from multiplying elements of fitness vectors per gene.

#### Remark 2.2

Both models, (8) and (19), are implemented in R Shiny and are available as a web-app at https://shiny.maths.nottingham.ac.uk/shiny/mutsel/. A description of the web-app is also available in Howitt (2018).

### Long-Time Behaviour of the Models

In this section, we study long-time behaviour of models (8) and (19). We start with model (8). Owing to the fact that model (8) resembles a linear Markov chain, we can study the limit of the distribution $\pi (n;\pi (0))$ as $n\rightarrow \infty$ using the standard theory of ergodic Markov chains (see, e.g. Meyn and Tweedie 2009) and prove the following proposition using the fact that the corresponding Markov chain has a finite number of states and under Assumption 2.2 all the elements of the matrix of transitional probabilities *T* are strictly positive.

#### Proposition 2.1

Let Assumption 2.2 hold. Then, when $n\rightarrow \infty ,$ the distribution $\pi (n;\pi (0))$ has the unique limit $^{\infty }\pi$ which is independent of $\pi (0)$ and is equal to

27 $$\begin{aligned} ^{\infty }\pi =\ ^{\infty }\pi _{1}\otimes \cdots \otimes \ ^{\infty }\pi _{\ell }, \end{aligned}$$

where $^{\infty }\pi _{i}$ are stationary distributions for single genes *i* and

$$\begin{aligned} ^{\infty }\pi _{i}^{1}=\frac{q_{i}}{p_{i}+q_{i}},\ \ ^{\infty }\pi _{i}^{2}= \frac{p_{i}}{p_{i}+q_{i}}. \end{aligned}$$

The proof of (27) is elementary and hence omitted here.

We also note that by standard results (see, e.g. Meyn and Tweedie 2009) $$\pi (n;\pi (0)) $$ converges to $$^{\infty }\pi $$ exponentially. The number of time steps $$ n_{s}$$ needed for $$\pi (n;\pi (0))$$ to reach a proximity of $$^{\infty }\pi ,$$ i.e. that for some $$\varepsilon >0$$ we have $$||\ ^{\infty }\pi -\pi (n;\pi (0))||\le \varepsilon ,$$ can be estimated (Bayliss et al. 2012) as

28 $$\begin{aligned} n_{s}\approx \frac{\ln \left( \varepsilon /||\ ^{\infty }\pi -\pi (n;\pi (0))||\right) }{\ln \max _{1\le i\le \ell }\left( 1-p_{i}-q_{i}\right) }, \end{aligned}$$

where $||\cdot ||$ is, e.g. the total variation norm.

Now let us discuss the mutation–selection model (19). Using Proposition 1.2 from Kolokoltsov (2010, Ch. 1), it is not difficult to prove the following proposition.

#### Proposition 2.2

Let Assumption 2.2 hold. Then, when $n\rightarrow \infty ,$ the distribution $\pi _{\text {sel}}(n;\pi (0))$ has a limit $^{\infty }\pi _{\text {sel}}$ for any initial $\pi (0).$

The next proposition is on uniqueness of the limit $^{\infty }\pi _{\text {sel}}$ independent of initial $\pi (0).$

#### Proposition 2.3

Let Assumption 2.2 hold. Assume that there is a positive constant $c<1$ and a number of steps $n\ge 1$ such that for any initial distributions $\breve{\pi }$ and $\tilde{\pi }:$

29 $$\begin{aligned} \left| \pi _{\text {sel}}(n;\breve{\pi })-\pi _{\text {sel}}(n;\tilde{\pi } )\right| _{1}\le c\left| \breve{\pi }-\tilde{\pi }\right| _{1}. \end{aligned}$$

Then, the limit $^{\infty }\pi _{\text {sel}}$ is unique.

#### Proof

By Proposition 2.2 for any initial distribution $$\pi (0),$$$$\pi _{\text {sel}}(n;\pi (0))$$ tends to a limit $$ ^{\infty }\pi _{\text {sel}}$$ as $$n\rightarrow \infty .$$ Suppose there are two different limits $$^{\infty }\breve{\pi }_{\text {sel}}$$ and $$^{\infty } \tilde{\pi }_{\text {sel}}$$ corresponding to two different initial distributions. We have $$\pi _{\text {sel}}(n;\ ^{\infty }\breve{\pi }_{\text { sel}})=\ ^{\infty }\breve{\pi }_{\text {sel}}$$ and $$\pi _{\text {sel}}(n;\ ^{\infty }\tilde{\pi }_{\text {sel}})=$$$$^{\infty }\tilde{\pi }_{\text {sel}}$$ for any *n*. From this and (29), we get

$$\begin{aligned} \left| \ ^{\infty }\breve{\pi }_{\text {sel}}-\ ^{\infty }\tilde{\pi }_{ \text {sel}}\right| _{1}=\left| \ \pi _{\text {sel}}(n;\ ^{\infty } \breve{\pi }_{\text {sel}})-\ \pi _{\text {sel}}(n;\ ^{\infty } \tilde{\pi }_{\text {sel}})\right| _{1}<\left| \ ^{\infty }\breve{\pi }_{\text {sel} }-\ ^{\infty }\tilde{\pi }_{\text {sel}}\right| _{1} \end{aligned}$$

which is not possible, and hence the limit is unique. Proposition 2.3 is proved. $\square$

#### Remark 2.3

We have not succeeded in showing that (29) holds for arbitrary parameters of model (19) but for each particular choice of the parameters *p*,  *q*,  $\gamma$ it is possible to verify (29) numerically by solving the constrained optimization problem to find the upper bound:

$$\begin{aligned} \sup _{\begin{array}{c} \breve{\pi },\tilde{\pi }\in \mathcal {E} \\ \breve{\pi }\ne \tilde{\pi } \end{array}}\frac{\left| \pi _{\text {sel}}(n;\breve{\pi })-\pi _{\text { sel}}(n;\tilde{\pi })\right| _{1}}{\left| \breve{\pi }-\tilde{\pi } \right| _{1}}, \end{aligned}$$

where $$\mathcal {E}=\{\pi :$$$$\left| \pi \right| _{1}=1$$ and all components of $$\pi $$ are non-negative$$\}$$. To solve this optimization problem, one can, e.g. use the function fmincon in MATLAB or the nloptr package in R. In all tests we did for particular sets of parameters, condition (29) was satisfied.

We note that condition (29) with $n=1$ (i.e. continuity of the mapping $\varPhi (\pi )$ [see (23)] with Lipschitz constant less than 1) is used in Butkovsky (2014) to prove uniqueness of invariant measure for nonlinear Markov chains in a general setting. But this condition is rather restrictive, e.g. it does not hold for our model (19) even in the case of a single gene when $1-p-q$ is positive and close to 1, $\gamma _{i}^{1}=1$ and $\gamma _{i}^{2}>1$.

#### Remark 2.4

In the case of a single gene, $\ell =1,$ the uniqueness of the limit $^{\infty }\pi _{\text {sel}}$ under Assumption 2.2 follows from Lemma A.1 in the Appendix.

In the general case, we were not able to find an explicit expression for $^{\infty }\pi _{\text {sel}}$ but we obtained such an expression in the case when Assumption 2.3 holds, which is given in Proposition 2.4 below. In the general case, the stationary distribution $^{\infty }\pi _{\text {sel}}$ for a particular set of parameters *p*,  *q*,  $\gamma$ can be found numerically by solving the system of $2^{l}-1$ quadratic equations.

#### Proposition 2.4

Let Assumptions 2.2 and 2.3 hold. Then, there is a stationary distribution $^{\infty }\pi _{\text {sel}}$ of the form

30 $$\begin{aligned} ^{\infty }\pi _{\text {sel}}=\ ^{\infty }\pi _{\text {sel,}1}\otimes \cdots \otimes \ ^{\infty }\pi _{\text {sel,}\ell }, \end{aligned}$$

where $\ ^{\infty }\pi _{\text {sel,}i}$ are stationary distributions for single genes *i* individually described by (19) and

$$\begin{aligned} ^{\infty }\pi _{\text {sel,}i}^{1}= & {} \dfrac{2\gamma _{i}^{1}q_{i}}{ (1-q_{i})\varDelta \gamma _{i}+\gamma _{i}^{1}(p_{i}+q_{i})+\sqrt{(\gamma _{i}^{1}p_{i}+\gamma _{i}^{2}q_{i})^{2}+2(\gamma _{i}^{1}p_{i}-\gamma _{i}^{2}q_{i})\varDelta \gamma _{i}+\left( \varDelta \gamma _{i}\right) ^{2}}}\ ,\\ ^{\infty }\pi _{\text {sel,}i}^{2}= & {} 1-\ ^{\infty }\pi _{\text {sel,}i}^{1}\ ,\ \varDelta \gamma _{i}=\gamma _{i}^{2}-\gamma _{i}^{1}\ . \end{aligned}$$

The proof of this proposition is given in “Appendix A”.

Result (30) has the interpretation that (assuming that the conditions of Proposition 2.3 are verified) in the stationary regime genes behave independently. It also means that if the initial population distribution $\pi (0)$ is such that genes behave independently then they do so for all times. Further, if the initial population distribution $\pi (0)$ is such that genes behave dependently then the strength of dependence decays with time. We know that often in practice (see, e.g. Woodacre et al. 2019; Lango-Scholey et al. 2019; Howitt 2018) this type of evolution behaviour is not the case, which demonstrates a limitation of model (19), (25) in being capable of explaining experimental data. At the same time, the mutation–selection model (19) does not have this deficiency.

#### Remark 2.5

The web-app from Remark 2.2 also gives $^{\infty }\pi$ and an accurate approximation of $^{\infty }\pi _{\text {sel}}.$

## Verifying Whether Data can be Explained by the Mutation Model

Typically (see, e.g. Bayliss 2009; Bayliss et al. 2012; Woodacre et al. 2019; Lango-Scholey et al. 2019), the following data are available from experiments aimed at understanding bacteria population genetics:

1. Estimates $$\hat{p}_{i},$$$$\hat{q}_{i}$$, $$i=1,\ldots ,\ell ,$$ of the mutation rates together with $$95\%$$ confidence intervals $$[\ _{*}p_{i},p_{i}^{*}]$$ and $$[\ _{*}q_{i},q_{i}^{*}],$$ respectively;
2. Average number of generations $\bar{n}_{k}$ between the time points $k-1$ and *k* together with the lowest possible $_{*}n_{k}$ and the largest possible $n_{k}^{*}$ number of generations;
3. Sample distributions of phasotypes $_{k}\hat{\pi }$ at time observation points $k=1,2,\ldots$ and sizes $N_{k}$ of the samples.

Estimates $$\ \hat{p}_{i},$$$$\hat{q}_{i}$$ of the mutation rates together with their confidence intervals are found during specially designed experiments (see, e.g. Bayliss 2009; Bayliss et al. 2012; Aidley and Bayliss 2014 and references therein). They are of order $$ 10^{-5}-10^{-2} $$ (Saunders et al. 2003; Bayliss et al. 2012). The mutation rates are estimated for repeat numbers and then mapped to phasotypes (see details in Bayliss et al. 2012 and also Howitt 2018). Note that these PV mutation rates are higher than those for genes which are non-phase variable. It is assumed (Saunders et al. 2003; Bayliss et al. 2012) that the mutation rates stay the same in all in vitro or in vivo experiments with this bacterium species.

The average number of generations $\bar{n}_{k}$ is computed by multiplying calendar time between the observation points by average division rate of the bacteria species being considered. The average division rate depends on the experimental conditions. Similarly, $_{*}n_{k}$ and $n_{k}^{*}$ are found using the slowest and fastest division rates for the bacteria. They are introduced to compensate for the use of average division rate and to reflect the stochastic nature of bacterial division. For example, in in vitro *C. jejuni *experiments (Woodacre et al. 2019) the average division rate was taken as 20 per 3 days, slowest—10 and fastest—25 (see also growth rates in caecal material in Battersby et al. 2016).

Sample distributions of phasotypes $_{k}\hat{\pi }$ are derived from sample distributions of tract lengths of the PV genes under consideration (Bayliss 2009; Bayliss et al. 2012). The tract length (i.e. the repeat number) is determined by DNA analysis of bacterial material collected during in vitro or in vivo experiments (see further details, e.g. in Bayliss 2009; Bayliss et al. 2012; Howitt 2018; Woodacre et al. 2019; Lango-Scholey et al. 2019). The models and the data assimilation procedures in this paper are aimed at understanding how a bacteria population evolves during a particular experimental setting via looking at time evolution of $_{k}\hat{\pi }$. We note that fitness parameters cannot be measured during a biological experiment.

Due to costs of conducting DNA analysis of bacteria, sample sizes $N_{k}$ are usually not big [e.g. of order $30-300$ (Bayliss et al. 2012; Woodacre et al. 2019; Lango-Scholey et al. 2019)]. Hence, $_{k}\hat{\pi }$ have a sampling error which cannot be ignored. Let us assume that if $N_{k}\rightarrow \infty$ then $_{k}\hat{\pi }$ converges to a distribution $_{k}\bar{\pi },$ i.e. from the practical perspective, if we get data for a very large sample then the statistical error is effectively equal to zero.

As discussed at the end of Sect. 2.1, we can check for each gene individually [see (15)] whether its behaviour can be explained by the mutation model, and hence determine a subset of PV genes [for *C. jejuni* strain NCTC11168, there are 28 known PV genes (Bayliss 2009; Bayliss et al. 2012)] for which evolution can be explained by the mutation mechanism alone. For the other genes, i.e. those which fail this test, an alternative model [e.g. (19)] should be used. Thus, we will consider in this section how to determine whether model (15) is consistent with data for a single gene.

To simplify exposition of the remaining part of this section, we will drop indices specifying a particular gene in the notation since we will work with a single gene. More precisely, we will use

- $$\pi =(\pi ^{1},\pi ^{2}),$$$$_{k}\hat{\pi }=(_{k}\hat{\pi }^{1},_{k}\hat{ \pi }^{2})$$ and $$_{k}\bar{\pi }=(_{k}\bar{\pi }^{1},_{k}\bar{\pi }^{2})$$ instead of $$\pi _{i},$$$$_{k}\hat{\pi }_{i}$$ and $$_{k}\bar{\pi }_{i},$$ respectively;
- *p*, *q*,  $$p_{*},$$$$p^{*},$$$$q_{*},$$$$q^{*}$$ instead of $$p_{i}$$, $$q_{i},$$$$_{*}p_{i},$$$$p_{i}^{*},$$$$_{*}q_{i},$$$$ q_{i}^{*},$$ respectively.

Further, since we will be working with a single time period, we only have time points $k=0$ and $k=1$ and we can simplify the notation as

- $$\bar{n}$$, $$n_{*},$$$$n^{*}$$ instead of $$\bar{n}_{1},$$$$_{*}n_{1},$$$$n_{1}^{*}.$$

Note that this simplification of notation applies only to the remainder of this section.

To quantify the distance between the two distributions, $_{k}\hat{\pi }$ and $_{k}\bar{\pi },$ we use the total variation distance:

31 $$\begin{aligned} ||\ _{k}\bar{\pi }-\ _{k}\hat{\pi }||_{TV}=|\ _{k}\bar{\pi }^{1}-\ _{k}\hat{\pi } ^{1}|. \end{aligned}$$

Conservatively (Noether 1963), we can estimate the above error using the one-sided Kolmogorov–Smirnov test with $95\%$ confidence level as

32 $$\begin{aligned} ||\ _{k}\bar{\pi }-\ _{k}\hat{\pi }||_{TV}\le \varepsilon _{k}:=\frac{1.2238}{ \sqrt{N_{k}}}\ . \end{aligned}$$

One can use more accurate estimates for the sample error, e.g. exploiting the Hellinger distance together with $\chi ^{2}$ test (Pitman 1979), but we use here the total variation distance for the sake of simplicity of the algorithm. Inequality (32) implies that with 95% probability

33 $$\begin{aligned} _{k}\bar{\pi }^{1}\in [\ \min (0,\ _{k}\hat{\pi }^{1}-\varepsilon _{k}),\ \max (1,\ _{k}\hat{\pi }^{1}+\varepsilon _{k})]. \end{aligned}$$

We use the following to mean that model (15) is consistent with data. Let

$$\begin{aligned} _{i}\varepsilon _{*}=\max (0,\ _{i}\hat{\pi }^{1}-\varepsilon _{i}) \ \ \text { and}\quad _{i}\varepsilon ^{*}=\min (1,\ _{i}\hat{\pi }^{1}+\varepsilon _{i}). \end{aligned}$$

If there are $$p\in [p_{*},p^{*}],$$$$q\in [q_{*},$$$$ q^{*}],$$$$n\in [n_{*},$$$$n^{*}]$$ and $$\pi ^{1}(0)\in [\ _{0}\varepsilon _{*},\ _{0}\varepsilon ^{*}]$$ such that $$ \pi ^{1}(n;\pi (0))\in [\ _{1}\varepsilon _{*},\ _{1}\varepsilon ^{*}]$$, with $$\pi (n;\pi (0))$$ found by (15), then we say that the data can be explained by the model. Otherwise, model (15) is not consistent with data for that gene. We note that this test is conservative in the sense that we are using broad confidence intervals, and if we determine that the data cannot be explained by model (15), we say so with a large certainty.

### Algorithm

Now we proceed to deriving an algorithm to verify whether one gene data can be explained by model (15). By simple linear algebra, we obtain from (15):

34 $$\begin{aligned} \pi ^{1}(n;\pi (0))=\frac{q}{p+q}+\left( 1-p-q\right) ^{n}\left[ \pi ^{1}(0)- \frac{q}{p+q}\right] . \end{aligned}$$

It is convenient to introduce the change of variables

35 $$\begin{aligned} x:=\frac{q}{p+q},\ \ y:=\left( 1-p-q\right) ^{n}. \end{aligned}$$

Using these new variables, we rewrite (34) as

36 $$\begin{aligned} \pi ^{1}(n;\pi (0))-x=y\left[ \pi ^{1}(0)-x\right] . \end{aligned}$$

In what follows, we will make the following biologically justified assumption (recall that PV mutation rates are of order $$10^{-5}-10^{-2}).$$

#### Assumption 3.1

Assume that $$0<p+q<1.$$

We see that under Assumption 2.2

37 $$\begin{aligned} x\in \mathbb {I}_{x}:=\left[ \frac{q_{*}}{p^{*}+q^{*}},\frac{ q^{*}}{p_{*}+q_{*}}\right] \subset (0,1) \end{aligned}$$

and under Assumption 3.1

38 $$\begin{aligned} y\in (0,1). \end{aligned}$$

**Fig. 1:**
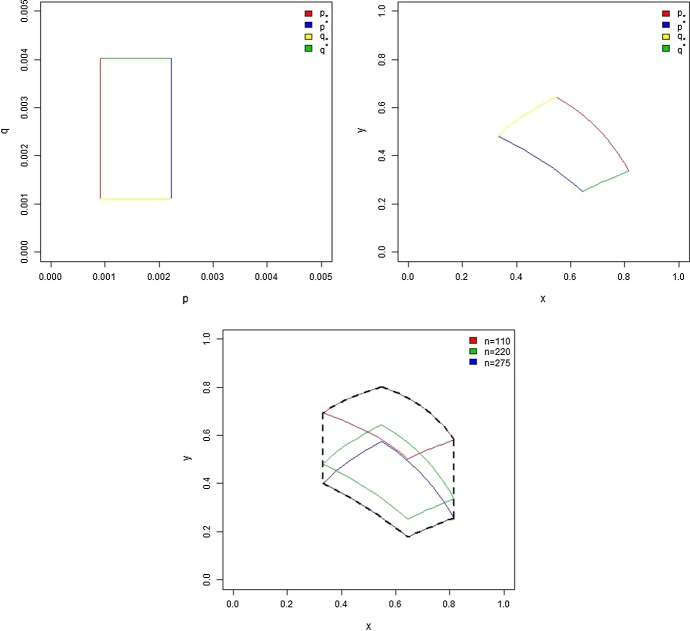
The domain $\mathbb {J}_{n}$ (top right), which is obtained from the (*p*, *q*) domain (top left), and the corresponding example of the domain $\mathbb {J}$ (bottom) (Color figure online)

For a fixed *n*,  (35) defines a map from (*p*, *q*) to (*x*, *y*). Let $$ \mathbb {J}_{n}$$ be a domain on the plane (*x*, *y*) obtained by this map applied to the rectangular domain $$[p_{*},p^{*}]\times [q_{*},$$$$q^{*}].$$ We also introduce a domain $$\mathbb {J}$$ on the plane (*x*, *y*) which is the minimal connected closed domain containing all $$ \mathbb {J}_{n},$$$$n\in [n_{*},$$$$n^{*}].$$ The map and the domains $$\mathbb {J}_{n}$$ and $$\mathbb {J}$$ are illustrated in Fig. 1. Now the question whether model (15) is consistent with data for a single gene can be reformulated using the new variables: if there is $$(x,y)\in \mathbb {J}$$ so that for $$u\in [\ _{0}\varepsilon _{*},\ _{0}\varepsilon ^{*}]$$ and $$v\in [\ _{1}\varepsilon _{*},\ _{1}\varepsilon ^{*}]$$ the equation

39 $$\begin{aligned} v-x=y\left[ u-x\right] \end{aligned}$$

has a solution, then the data can be explained by model (15). To answer this question, we formulate the algorithm below in which the outcome “Yes” means that model (15) is consistent with given single-gene data and “No” means not consistent.

**Algorithm 3.1** Given single-gene data, compute $$ _{i}\varepsilon _{*},$$$$_{i}\varepsilon ^{*}$$, $$i=1,2,$$$$\mathbb {I} _{x}$$ and $$\mathbb {J}$$.

Step 1 If there are $$x\in \mathbb {I}_{x},$$$$u\in [\ _{0}\varepsilon _{*},\ _{0}\varepsilon ^{*}]$$ and $$v\in [\ _{1}\varepsilon _{*},\ _{1}\varepsilon ^{*}]$$ such that $$x=u=v$$ then Yes, otherwise go to Step 2.
Step 2 For all $x\in \mathbb {I}_{x}$ and $u\in [\ _{0}\varepsilon _{*},\ _{0}\varepsilon ^{*}]$ such that $x\ne u$, and for $v\in [\ _{1}\varepsilon _{*},\ _{1}\varepsilon ^{*}],$ form the parametrized set of functions
  40 $$\begin{aligned} y(x;u,v)=\frac{v-x}{u-x}.\text { } \end{aligned}$$
  If for $x\in \mathbb {I}_{x}$ a curve (*x*, *y*(*x*; *u*, *v*)) with *y*(*x*; *u*, *v*) defined in (40) intersects the domain $\mathbb {J}$ then Yes; otherwise No.

We note that if the data satisfy the condition of Step 1 of the above algorithm then, in addition to the conclusion that model (15) can explain the data, it is also plausible that evolution of this gene can be stationary (i.e. the distribution is not changing with time).

#### Remark 3.1

Algorithm 3.1 verifying whether the data can be explained by the mutation model (8) is implemented in R Shiny and is available as a web-app at https://shiny.maths.nottingham.ac.uk/shiny/gene_algorithm/.

#### Remark 3.2

We note that we can verify whether one gene data can be explained by model (15) using an analogue of the ABC algorithms (Algorithms 4.1 and 4.2) from Sect. 4 in the same spirit as we answer this question in the case of the mutation–selection model (19) in Sects. 4 and 5. But ABC algorithms are more computationally expensive as they are sampling based, requiring the use of Monte Carlo techniques, while Algorithm 3.1 is deterministic and very simple with negligible computational cost.

### Illustrations

We illustrate Algorithm 3.1 by applying it to the data for three (*cj*0617,  *cj*1295 and *cj*1342) out of 28 PV genes obtained in in vitro experiments (Woodacre et al. 2019) (see also Howitt 2018). Statistical analysis of the two genes done in Woodacre et al. (2019) and Howitt (2018) suggested that *cj*0617 is a part of a small network of dependent genes, and hence it is likely to be subject to selection, while both *cj*1295 and *cj*1342 did not demonstrate any dependencies with the other 27 PV genes, and hence they are likely to have evolution which can be explained by the mutation mechanism alone.

The data for these three genes are as follows (Woodacre et al. 2019; Howitt 2018):

*cj*0617: $$_{0}\hat{\pi }^{1}=0.943$$, $$_{1} \hat{\pi }^{1}=0.262$$, $$p_{*}=9.1\times 10^{-4},\ p^{*}=22.2\times 10^{-4}$$, $$q_{*}=11.0\times 10^{-4},$$$$q^{*}=40.2\times 10^{-4}$$, $$ n_{*}=110,$$$$n^{*}=275$$, $$N_{0}=300$$, $$N_{1}=145$$.
*cj*1295: $$_{0}\hat{\pi }^{1}=0.305$$, $$_{1}\hat{\pi } ^{1}=0.174$$, $$p_{*}=3.0\times 10^{-4},\ p^{*}=5.7\times 10^{-4},$$$$ q_{*}=1.4\times 10^{-4},$$$$q^{*}=2.8\times 10^{-4},$$$$n_{*}=110,$$$$n^{*}=275$$, $$N_{0}=298$$, $$N_{1}=149$$.
*cj*1342 :  $$_{0}\hat{\pi }^{1}=0.017$$, $$_{1} \hat{\pi }^{1}=0.153$$, $$p_{*}=11.0\times 10^{-4},\ p^{*}=40.2\times 10^{-4},$$$$q_{*}=9.1\times 10^{-4},$$$$q^{*}=22.2\times 10^{-4},$$$$ n_{*}=110,$$$$n^{*}=275$$, $$N_{0}=298$$, $$N_{1}=150$$.

Therefore, for *cj*0617 we have $$\mathbb {I}_{x}=[0.331,0.815]$$, $$\varepsilon _{0}=0.071$$,  $$\varepsilon _{1}=0.102$$, and hence $$ _{0}\varepsilon _{*}=0.872,\,_{0}\varepsilon ^{*}=1,$$$$ _{1}\varepsilon _{*}=0.160,$$$$_{1}\varepsilon ^{*}=0.364$$; for *cj*1295 we have $$\mathbb {I}_{x}=[0.197,0.517]$$, $$\varepsilon _{0}=0.071$$, $$ \varepsilon _{1}=0.10,$$ and hence $$_{0}\varepsilon _{*}=0.234,$$$$ _{0}\varepsilon ^{*}=0.376,$$$$_{1}\varepsilon _{*}=0.074$$, $$ _{1}\varepsilon ^{*}=0.274$$; and for *cj*1342 we have $$\mathbb {I} _{x}=[0.185,0.669],$$$$\varepsilon _{0}=0.071$$, $$\varepsilon _{1}=0.10,$$ and hence $$_{0}\varepsilon _{*}=0,$$$$_{0}\varepsilon ^{*}=0.088,$$$$_{1}\varepsilon _{*}=0.053,$$$$_{1}\varepsilon ^{*}=0.253.$$

**Fig. 2:**
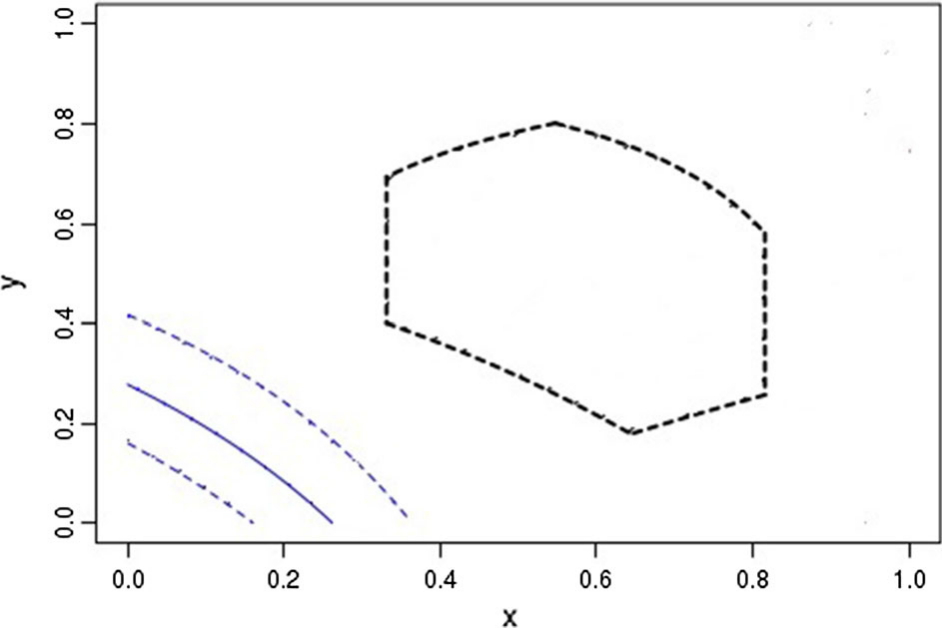
Application of Algorithm 3.1 to the data for gene *cj*0617. The domain $\mathbb {J}$ is shown by black dashed lines; the blue dashed curves are $y_{\min }(x)$ and $y_{\max }(x)$; the solid blue curve is $y(x;\ _{0} \pi ^1, \ _{1}\pi ^1)$ (Color figure online)

Application of Algorithm 3.1 to the data for *cj*0617 gene gives us:

Step 1 Since $[0.331,0.815]\cap [0.872,\ 1]\cap [0.160,0.364]=\varnothing ,$ we get No and we go to Step 2.
Step 2 We have under $$x\in [0.331,0.815],$$$$u\in [0.872,\ 1],$$ and $$v\in [0.160,0.364]$$:
  $$\begin{aligned} y_{\min }(x)\le y(x;u,v)\le y_{\max }(x), \end{aligned}$$
  where
  $$\begin{aligned} y_{\min }(x)=\frac{0.160-x}{1-x}\text { and }y_{\max }(x)=\frac{0.364-x}{ 0.872-x}, \end{aligned}$$
  and we observe in Fig. 2 that the curves $(x,y_{\min }(x))$ and $(x,y_{\max }(x))$ do not intersect the domain $\mathbb {J,}$ and hence we conclude that the mutation model cannot describe evolution of this gene.

Application of Algorithm 3.1 to the data for *cj*1295 gene gives us

Step 1 Since $[0.197,0.517]\cap [0.234,0.376]\cap [0.074,0.274]\ne \varnothing ,$ we conclude that this gene can be described by the mutation model and it is possible that its evolution is stationary.

Application of Algorithm 3.1 to the data for *cj*1342 gene gives us

**Fig. 3:**
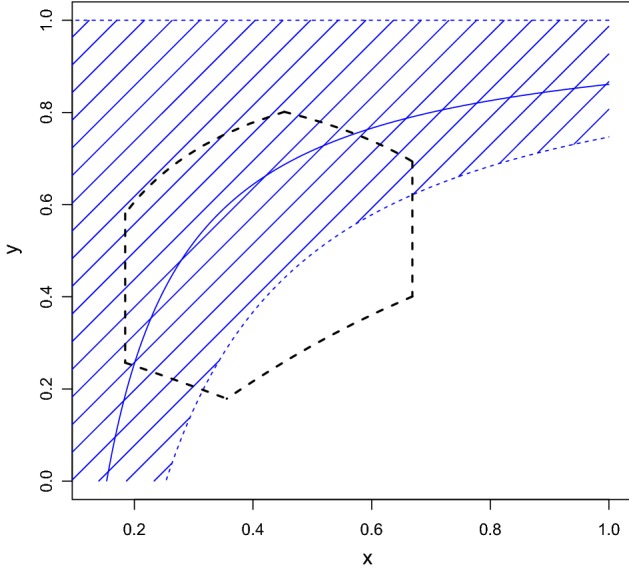
Application of Algorithm 3.1 to the data for *cj*01342 gene. The domain $\mathbb {J}$ is shown by black dashed lines; the blue dashed curve is $y_{\min }(x)$; the solid blue curve is $y(x; \ _{0}\pi ^1, \ _{1} \pi ^1)$. The blue cross-hatched region shows the domain covered by *y*(*x*; *u*, *v*) as described in the text (Color figure online)

Step 1 Since $[0.185,0.669]\cap [0,\ 0.088]\cap [0.053,0.253]=\varnothing ,$ we get No and we go to Step 2.
Step 2 We have under $$x\in [0.185,0.669],$$$$u\in [0,\ 0.088],$$ and $$v\in [0.053,0.253]$$:
  41 $$\begin{aligned} y_{\min }(x)\le y(x;u,v)\le 1, \end{aligned}$$
  where
  $$\begin{aligned} y_{\min }(x)=\frac{x-0.253}{x}\text { } \end{aligned}$$
  (the bounds in (41) are achievable) and observe in Fig. 3 that the curve $(x,y_{\min }(x))$ intersects the domain $\mathbb {J}$, and hence we conclude that the mutation model can describe evolution of this gene.

Further illustrations for Algorithm 3.1 are available in Howitt (2018).

## Estimation of Fitness Parameters in the Mutation–Selection Model

In this section, we describe our general methodology for the estimation of fitness parameters. We will illustrate the use of this methodology using data from *C. jejuni* experiments in Sect. 5. We adopt a Bayesian approach, whereby uncertainty in any unknown parameters is summarized by probability distributions. We illustrate how uncertainty in random quantities can be incorporated very naturally in the Bayesian framework, using prior information from previous experiments where available, and show how estimates in all quantities can be obtained in light of the observed data.

### Bayesian Statistics

In general terms, we have a sample of data *x* (realizations of a random variable *X*), whose distribution depends on some vector of parameters $\varTheta$. Upon adopting some probability model for the data-generating process, the likelihood function is $f_{X|\varTheta }(x|\theta )$, the distribution of *X* given $\varTheta$. In the Bayesian setting, the parameter $\varTheta$ is considered a random variable, and uncertainty in this parameter is initially described by a prior distribution, $f_{\theta }(\theta )$. Upon observing *x*, Bayes theorem gives

42 $$\begin{aligned} f_{\varTheta |X}(\theta |x) = \frac{f_{X|\varTheta }(x|\theta )f_{\theta }(\theta )}{ f_{X}(x)}, \end{aligned}$$

the posterior distribution of $\varTheta$ given *x*, which completely describes uncertainty in $\varTheta$ after learning *x*. The posterior distribution can then be used to compute any summaries of interest, such as probability intervals for components of $\varTheta$ or point estimates such as the mean of the posterior distribution. For ease of exposition, in what follows we will drop the subscripts denoting the random variable a distribution refers to, which is clear from the context. For example, we will simply write $f(\theta |x)$ for $f_{\varTheta |X}(\theta |x)$. For an account of Bayesian methodology with an emphasis on applications, see, e.g. Gelman et al. (2013) or Wilkinson (2012), where the latter has a biological focus.

Computing summaries from the posterior distribution requires integration, which in practice is not possible analytically except for simple models. One can adopt numerical procedures, but the performance of these degrades quite rapidly as the dimension of $\varTheta$ increases. A powerful alternative is to use simulation methods, which also have the major advantage of not requiring the normalizing constant *f*(*x*) in (42), the so-called marginal likelihood, which again requires an integration which is typically computationally expensive. If one can draw independent samples directly from $f(\theta |x)$, then Monte Carlo techniques can be used to estimate posterior quantities of interest. For complex, typically high-dimensional, models, this itself may be difficult, but powerful techniques such as Markov chain Monte Carlo (MCMC) can be employed (Gelman et al. 2013; Gilks et al. 1996; Wilkinson 2012). MCMC itself can be difficult to implement effectively in some complex scenarios, and it can be computationally demanding. An important recent development is the Integrated Nested Laplace Approximation (INLA) method (Rue et al. 2009), which as the name suggests, is based on Laplace approximations to the required integrals. The Laplace method itself is a well-known tool for approximating integrals in general (de Bruijn 1981) and has been used effectively in Bayesian statistics to compute posterior summaries (Tierney and Kadane 1986). INLA extends this idea to models with a general latent Gaussian structure and allows comparatively fast and simple approximations, which can either be used as an alternative to, or in conjunction with, simulation methods such as MCMC.

However, a further complication, which arises in our case, is that it may not even be possible to evaluate the likelihood $f(x|\theta )$, which is necessary for the simulation methods mentioned above. In this case, so-called likelihood-free methods can be employed, an example of which is Approximate Bayesian Computation (ABC) (Beaumont 2010), which we use here. This assumes the ability to simulate from the model $f(\cdot |\theta )$ relatively easily, even if evaluation of the likelihood itself is not possible.

### Approximate Bayesian Computation

Suppose it is straightforward to sample from $f(x|\theta )$, but evaluation of $f(x|\theta )$ itself is not possible. Recall that the objective is to simulate samples from $f(\theta |x)$, in order to perform Monte Carlo inference about $\theta$. This can be done via the following algorithm (Beaumont 2010):

1. Simulate $$\theta \sim f(\theta )$$;
2. Simulate $$y \sim f(x|\theta )$$;
3. Accept $\theta$ if $y=x$, else return to step 1.

This returns a sample $\theta$ from $f(\theta |x)$, and the process can be repeated until the desired number of samples is obtained. However, if the data are continuous and/or high-dimensional, then the event $y=x$ in the above algorithm will occur with zero, or very small, probability. Hence, in most practical situations, the condition that $y=x$ is replaced with the condition that $d(x,y) \le \epsilon$, for some distance function *d* and tolerance $\epsilon >0$. Hence, accepted samples $\theta$ are not from the exact posterior distribution of interest, but from some approximation $\tilde{f}(\theta |x)$ to the true posterior distribution. Informally, we would expect that the approximation is better the smaller the value of $\epsilon$, and under quite mild conditions, Monte Carlo estimators of posterior quantities converge to unbiased estimators as $\epsilon \rightarrow 0$ (Barber et al. 2015).

### General Algorithm

As discussed in Sect. 3, our data are the observed sample phasotype distributions $_{i}\hat{\pi }$, where $i=0$ is the initial timepoint and $i=1$ is the final timepoint. Our main question of interest is whether the proposed mutation–selection model (19) can explain the observed data, that is, are there values of the unknown quantities which are both biologically plausible and for which the final distribution obtained by model (19) is consistent with the observed sample? Recall that model (19) has input parameters $\theta =(n,p,q,_{0}\hat{\pi },\gamma )$, where *n* is the number of generations, *p* and *q* are the vectors of mutation rates, $_{0}\pi$ is the initial distribution, and $\gamma$ is the vector of fitness parameters. In general, we will treat all elements of $\theta$ as random, and we write $\varTheta =(\eta ,P,Q,_{0}\varPi ,\varGamma )$ for the corresponding random vector. Then, in general, the random variables are the elements of $\varTheta$ together with the final distribution $_{1}\varPi$ (a realization of which we denote by $_{1}\pi$); here, $_{1}\varPi$ plays the role of *X* in (42), i.e. the output of the probabilistic model.

Considering first all quantities other than $\varGamma$ to be fixed, another way to phrase our main question is: is there a value of $\varGamma$ for which the final distribution obtained from model (19) is “close to” the observed sample final distribution? In this case, there would be no evidence to reject the hypothesis that our proposed model is a plausible description of the evolution of phasotypes. The estimate of $\varGamma$ is also of interest in its own right, for biologists to understand which phasotypes or genes benefit from advantageous selection.

While there may be estimates or observations of the various quantities we consider random, there is often uncertainty. For instance, in our applications discussed in Sect. 5, there are estimates and plausible ranges available for *P*, *Q* and $\eta$. For the observed sample distributions $_i\hat{\pi }$, we have only a relatively small sample from a larger population, and hence our observations are subject to sampling variation. In both cases, uncertainty can be handled very naturally in the Bayesian framework, by encoding our existing knowledge in prior distributions. Our question then becomes: while accounting for uncertainty in all unknown quantities, can the mutation–selection model explain the evolution of phasotypes given our observed data?

Let $f(\theta )=f(n)f(p)f(q)f(_{0}\pi )f(\gamma )$ be the prior distribution on $\varTheta$. Thus, we assume independence between these quantities a priori, and we also assume that the elements of *P*, *Q* and $\varGamma$ are all mutually independent so that, e.g. $f(p_{1},\ldots ,p_{l})=f(p_{1})\ldots f(p_{l})$, etc. This independence assumption for the prior is natural from the microbiology point of view.

The prior distributions we use and the methods for sampling from them are discussed below. Assuming for now that we can simulate from these priors, then Algorithm 4.1 gives the steps taken to simulate from the ABC posterior distribution. We write $\pi _{\text {sel}}(\theta )$ for the output of the mutation–selection model (19), replacing $(n,p,q,_{0}\pi ,\gamma )$ with $\theta$.

**Algorithm 4.1** (*ABC algorithm for the mutation–selection model*)

Step 1 Propose a candidate value $\theta ^*\sim f(\theta )$.
Step 2 Obtain $\pi _{\text {sel}}(\theta ^*)$ by mutation–selection model (19).
Step 3 Accept $\theta ^*$ if $d(_1\hat{\pi },\pi _{\text { sel}}(\theta ^*))\le {}_1\epsilon$, where *d* is a distance function and $_1\epsilon$ is a tolerance. Otherwise, discard $\theta ^*$.

Steps 1–3 are then repeated until the desired number of samples from (the approximation to) the posterior distribution $f(\theta |x)$ is obtained. The choices of *d* and $_{1}\epsilon$ are discussed below.

The samples can then be used to form Monte Carlo estimates of the required quantities. In our applications, we use the mean of the samples to form point estimates and denote the estimates by $\hat{\gamma }$, etc. When accounting for sampling variability in the initial sample distribution, we denote an estimate of the true population distribution by $_0 \hat{\dot{\pi }}$ (to distinguish this from the observed sample which we denote by $_0\hat{\pi }$)—this is the (normalized) element-wise mean of the sampled initial distributions. To quantify uncertainty in the estimated parameters, we give $95\%$ posterior probability intervals; these are simply the $2.5\text {th}$ and $97.5\text {th}$ percentiles of the accepted samples, which are estimates of the true percentiles of the (marginal) posterior distribution for a given parameter.

Note that, in terms of model (19) itself, there is a certain non-identifiability surrounding the fitness parameters, since $\gamma$ and $k\gamma$, for some $k >0$, give the same model. Recall from Sect. 2.2 that we interpret the fitness parameters as relative fitness and remove this non-identifiability by taking the smallest fitness parameter to be 1, which is natural. In all our simulations, normalization is applied at the final stage. Specifically, let $\hat{\gamma }^*$ be an unnormalized vector, formed by taking the element-wise mean of all sampled fitness vectors (which are themselves un-normalized). Then, we set $\hat{ \gamma } = \hat{\gamma }^*/k$, where $k = \min (\hat{\gamma }^*)$, so that $\hat{\gamma }$ is the required estimate of relative fitness parameters.

### Simulation from Priors

In general, prior distributions are chosen which reflect the current knowledge about the unknown parameters. Here, we illustrate the choice of priors we use in our applications, but other prior distributions could be used when relevant.

*Fitness parameters* As discussed in Sect. 2.2 , the quantities of interest are the relative fitness parameters $\gamma$. We assign independent uniform priors to the fitness parameters, i.e. $\gamma ^i \sim U[a_i,b_i], \,i=1,\ldots ,2^l$, where $a_i \ge 1$, since $\gamma = 1$ for the slowest growing phasotype (see Sect. 2.2).

*Number of generations* For the number of generations $\eta$, we have from microbiology knowledge (see Sect. 3) an estimate $\bar{n}$ and interval $[n_{*},n^{*}]$ in which $\eta$ lies. The interval $[n_{*},n^{*}]$ is typically not symmetric around $\bar{n}.$ We construct a prior for $\eta$ from a skew-normal distribution, with mean $\bar{n}$, such that $P(n_{*}-\frac{1}{2}\le \eta \le n^{*}+\frac{1}{2})=0.95$—this is then discretized to give a probability mass function, since $\eta$ is integer-valued.

*Mutation rates* For the mutation rates *p* and *q*, as with the number of generations, there are estimates ($$\bar{p}$$ and $$\bar{q}$$ ) and $$95\%$$ confidence intervals available ($$[p_*,p^*]$$ and $$ [q_*,q^*]$$) from specially designed experiments (Bayliss et al. 2012). We form analogous prior distributions for these quantities via the same process as for $$\eta $$, minus the discretization as these quantities are continuous.

*Observed sample distributions* We account for sampling variability in distributions using probabilistic results for the distribution of distances. Specifically, we use the Hellinger distance to measure distance between two probability distributions and use the relationship between this distance and the $\chi ^2$ distribution to ascertain plausible discrepancies between two distributions if they are still to be considered the same after accounting for statistical variation.

The Hellinger distance between two discrete probability distributions $\phi _0$ and $\phi _1$ over a finite sample space $\varOmega$ is

43 $$\begin{aligned} H(\phi _0,\phi _1)= & {} \frac{1}{\sqrt{2}} \left\| \sqrt{\phi _0}-\sqrt{ \phi _1}\right\| _2 \nonumber \\= & {} \frac{1}{\sqrt{2}} \sqrt{\displaystyle \sum _{x \in \varOmega } \left( \sqrt{ \phi _0(x)} - \sqrt{\phi _1(x)}\right) ^2}, \end{aligned}$$

where $||\,\,. \,\,||_2$ is the Euclidean metric and $\phi _i(x) = P(X=x)$ if random variable $X \sim \phi _i$.

Now, let $\phi _0$ be a specified discrete probability distribution, corresponding to a random variable *X* with state space $\varOmega$ and $|\varOmega | = k < \infty$. Also, let $\phi _1$ be the empirical distribution formed from *N* realizations of *X*. Then,

44 $$\begin{aligned} 8NH^2(\phi _0,\phi _1) \sim \chi ^2_{k-1}, \end{aligned}$$

where $\chi ^2_{k-1}$ is the chi-squared distribution with $k-1$ degrees of freedom (Pitman 1979). Thus, one cannot reject the null hypothesis that the observed samples are from $\phi _0$, (at the significance level of $\alpha )$, if $8NH^2(\phi _0,\phi _1) < \chi ^2_{k-1}(1-\alpha )$, where $\chi ^2_{k-1}(1-\alpha )$ is the $100(1-\alpha )\%$ critical value of the $\chi ^2_{k-1}$ distribution. We use this relationship in reverse in order to obtain a tolerance $\epsilon$, where

45 $$\begin{aligned} \epsilon = \sqrt{\frac{\chi ^2_{k-1}(0.95)}{8N}}. \end{aligned}$$

Thus, if $H(\phi _0,\phi _1) < \epsilon$, there is no evidence to suggest that $\phi _1$ is statistically different to $\phi _0$ at the 0.05 significance level.

We also use this idea to account for sampling variability in an observed sample distribution $\hat{\phi }$, based on a sample size *N*, as follows. We first obtain a tolerance $\epsilon = \sqrt{\frac{\chi ^2_{k-1}(0.95)}{8N}}$, such that any distribution within (Hellinger) distance $\epsilon$ of $\hat{ \phi }$ defines a $95\%$ confidence region for the true population distribution $\phi$ of which $\hat{\phi }$ is an empirical estimate. We then construct a Dirichlet distribution, centred on $\hat{\phi }$, with parameter $\alpha = \alpha _0 \varvec{1}_{2^l}$, $\alpha _0 \in \mathbb {R}_+$, $\alpha \in \mathbb {R}_+^{2^l}$ such that $P(H(\varPhi ,\hat{\phi }) < \epsilon ) = 0.95$ where $\varPhi \sim \hbox {Dir}(\alpha )$. To account for sampling variability in the observed distribution, we sample an observation $\phi ^*$ from this Dirichlet distribution and accept $\phi ^*$ if $H(\phi ^*,\hat{\phi }) < \epsilon$ . Thus, we can think of an accepted $\phi ^*$ as a plausible sample distribution which could have been observed instead of $\hat{\phi }$.

Finally, we use the same procedure to obtain the tolerance used in the ABC algorithm (step 3 of Algorithm 4.1). Specifically, if the observed final distribution is based on a sample size of *N*, then the tolerance used is that given by (45).

### Dependence of Gene Fitness Parameters

Recall the earlier discussion in Sect. 2.2 regarding dependence between the selection/fitness parameters of different genes. Specifically, under the assumption of independence (Assumption 2.3), $\gamma$ is written as the tensor product (25). We introduce below an algorithm which can be used to test this assumption. In Sect. 5.1, we illustrate this on experimental data and show that the independence assumption does not hold for these data.

Recall that the fitness parameters for a gene *l* are $\gamma _l^1$ and $\gamma _l^2$, and $\gamma _l = (\gamma _l^1,\gamma _l^2)$. In short, we estimate the full vector of fitness parameters, $\gamma$, under the assumption of independence, and then assess whether the distance between the observed sample final distribution and that obtained from model (19), with $\gamma = \hat{\gamma }$, is less than the tolerance given by (45). This is detailed in Algorithm 4.2. Note that here we focus on how to handle the fitness parameters, and assume the other elements of $\theta$ are available—these could be fixed estimates, or estimated (with uncertainty incorporated) as part of steps 1 and 2 in Algorithm 4.2.

**Algorithm 4.2 **(*Verification of independence of fitness parameters*)

Step 1 Estimate $\gamma _l$, $l=1,\ldots ,\ell$ (and other elements of $\theta$ if required), using Algorithm 4.1 for each gene separately.
Step 2 Form $\hat{\gamma }^{\text {ind}} =\hat{\gamma }_{1}\otimes \cdots \otimes \hat{\gamma }_{\ell }$ and $\hat{\theta }$.
Step 3 Obtain the final distribution under the independence assumption, $\pi ^{\text {ind}}_{\text {sel}}(\hat{\theta })$, from (19).
Step 4 Compute $$d(_1\hat{\pi },\pi ^{\text {ind}}_{\text {sel} }(\hat{\theta }))$$.

Given a tolerance $_{1}\epsilon$, computed from (45), then there is evidence to reject the assumption of independent fitness per gene if $d(_{1}\hat{\pi },\pi _{\text {sel} }^{\text {ind}}(\hat{\theta }))>{}_{1}\epsilon$. This test is of obvious microbiological importance since if Assumption 2.3 is rejected, this means that selection acts on phasotypes rather than only on a state of a particular gene, i.e. that bacterial adaptation to the environment is regulated by a number of dependent genes.

## Results

We now illustrate our methodology with applications to data on the bacteria *C. jejuni*, using data from two in vitro experiments. Full experimental details for these experiments can be found in Woodacre et al. (2019) and also in Howitt (2018). We focus attention on three genes of interest, for which preliminary investigation has found evidence of dependent switching from one PV state to another (Woodacre et al. 2019; Howitt 2018). These genes are labelled *cj*0617, *cj*0685 and *cj*1437; note that the sample space of phasotypes is labelled according to the conventions described in Sect. 2 and Eq. (2), and in what follows, the ordering is with respect to the ordering of the genes as listed above. We first investigate whether the assumption of independence of fitness parameters is justifiable, using Algorithm 4.2, and show that there is evidence this assumption does not hold. We then illustrate the ability of our methodology to successfully estimate fitness parameters using synthetic data, before obtaining estimates of fitness parameters for our experimental data. We conclude this section with an experiment which provides evidence that switching of phasotypes occurs quickly when bacteria are subject to new environmental conditions, which suggests an interesting direction for future work involving time-dependent fitness parameters. Throughout this section, we used 500,000 Monte Carlo samples for all inferences based on ABC simulation, except for the single-gene results given in Table 1, which are based on 100,000 samples.

### Remark 5.1

Since we are only dealing with a relatively small number of genes, the ABC algorithm in the form proposed here is feasible in terms of computational complexity. As the dimension of the state space is $2^l$, then clearly the dimension of the parameter space grows exponentially with the number of genes, and it would not be practical to apply the ABC algorithm for many genes, say more than 6. However, we emphasize that our overall procedure is a two-stage process. Firstly, we reduce the number of genes on which to focus, by using the fast and efficient algorithm of Sect. 3 to determine which genes can be explained by the mutation model. Secondly, we then apply the mutation–selection model to the small number of remaining genes.

**Table 1:** Single-gene data, estimates and results for the independence of fitness parameters investigation

Gene	$_0\hat{\pi }$	$_1\hat{\pi }$	$\hat{\gamma }$
*cj*0617	(0.9433, 0.0567)	(0.2621, 0.7379)	(1, 1.016)
*cj*0685	(0.0567, 0.9433)	(0.8267, 0.1733)	(1.02, 1)
*cj*1437	(0.0533, 0.9467)	(0.8288, 0.1712)	(1.02, 1)

### Independence Assumption

In Table 1, we give the data for the single-gene runs of Algorithm 4.1, required in step 1 of Algorithm 4.2, and the (normalized) estimates $\hat{\gamma }_l,l=1,2,3$. In Table 2, we give the resulting input $\hat{\gamma }^{\text {ind}}$ for the three-gene model under Assumption 2.3, the corresponding output $\pi ^{\text {ind}}_{\text {sel} }(\hat{\theta })$, and the distance between the model output distribution and observed final distribution. In the same table, we also present the analogous results for the general model, i.e. when Algorithm 4.1 is applied to the three genes simultaneously, without applying Assumption 2.3—the fitness parameter estimates and model output are denoted $\hat{\gamma }^{ \text {gen}}$ and $\pi ^{\text {gen}}_{\text {sel}}(\hat{\theta })$, respectively. Note that throughout this subsection we have kept all quantities other than the fitness parameters fixed at their observed/estimated values. Also, other required quantities not in Tables 1 and 2 can be found in Tables 5 and 6, as explained in full in the caption to Table 2. The crucial observation is that, under the independence assumption, the distance between the observed final distribution and that predicted by the model using the estimated fitness parameters is greater than the tolerance allowing for ABC sampling error. In contrast, when Assumption 2.3 is relaxed, the distance is comfortably under the tolerance (see Table 2). We therefore reject the independence assumption here, and all the biological conclusions and interpretation which follow relate to results obtained using the more general model (19) without applying Assumption 2.3.

**Table 2:** Three-gene model input (fitness parameters) and results, with and without application of Assumption 2.3

$\hat{\gamma }^{\text {ind}}$	$\pi ^{\text {ind}}_{\text {sel}}(\hat{\theta })$	$\hat{\gamma }^{\text {gen}}$	$\pi ^{\text {gen}}_{\text {sel}}(\hat{\theta })$
(1.040400, 1.020000,	(0.099859, 0.000256,	(1.018, 1.007,	(0.143176, 0.011395,
1.020000, 1.000000,	0.002181, 0.000143,	1.009, 1.000,	0.009522, 0.056227,
1.057046, 1.036320,	0.877841, 0.000756,	1.026, 1.027,	0.685888, 0.033098,
1.036320, 1.016000)	0.018654, 0.000311)	1.019, 1.004)	0.036405, 0.024289)

Here, and for the single-gene results in Table 1, $p_l$, $q_l$ and *n* are fixed at the values $\bar{p}_l$, $\bar{q}_l$ and $\bar{n}$ given in Table 5, where the prior settings for the fitness parameters can also be found. The values of $_0N$ ($_0\epsilon$) and $_1N$ ($_1\epsilon$) required for the three-gene runs are as in Table 6. We obtain the distances $d(_1\hat{\pi }$,$\pi ^{\text {ind}}_{ \text {sel}}(\hat{\theta })) = 0.290$ and $d(_1\hat{\pi }$,$\pi ^{\text {gen}}_{ \text {sel}}(\hat{\theta }) = 0.067$; since $_1\epsilon = 0.112$, we reject the independence assumption

### Synthetic Data

Before analysing experimental data, we first test our inference procedure using synthetic data which mimic the data to be considered in Sect. 5.3 in important respects. Specifically, $_0\hat{\pi }$ and $\gamma$ were chosen such that the mutation–selection model produces a final distribution which is close to that observed in the real experimental data. We then assess our ability to recover $\gamma$. The sample data and prior settings are given in Table 3, except for the mutation rates *p* and *q*, for which the settings are the same as in Table 5. (Note that we use the same labelling of genes in our synthetic data as in the first experimental dataset of Sect. 5.3, since the synthetic data are constructed based on characteristics of the experimental data.) Upon obtaining our estimates for all random quantities, we use the mutation–selection model with these estimates as inputs to obtain the final distribution predicted by the model. The distance between the predicted and actual final distribution is 0.0457 (see Table 4), which in particular is less than the tolerance of 0.108 which allows for sampling error [from (45)]. The estimate $\hat{\gamma }$ is given in Table 4, which shows that it is close to the truth. From this we conclude that our inferential procedure is successful in recovering the true fitness parameters.

**Table 3:** Inputs for the synthetic data experiment

$_0N$	$_0\epsilon$	$_0\hat{\pi }$	$[a_i, b_i]$ for $\varGamma$	$_1N$	$_1\epsilon$	$_1\hat{\pi }$
300	0.0766	(0.003, 0.010, 0.007,	[1.005, 1.04]	150	0.108	(0.13013, 0.01044, 0.01129,
		0.924, 0.043,	[1, 1]			0.13676, 0.63192, 0.00608,
		0, 0, 0.013)	[1, 1]			0.03386, 0.03951)
			[1, 1]			
			[1.005, 1.04]			
			[1.005, 1.04]			
			[1.005, 1.04]			
			[1.005, 1.04]

**Table 4:** Results for the synthetic data experiment

True $\gamma$	$\hat{\gamma }$	$\pi _{\text {sel}}(\hat{\theta })$
(1.014, 1.002, 1.007, 1, 1.022	(1.0162, 1, 1, 1, 1.0252,	(0.12607, 0.00664, 0.00495, 0.11870,
1.01, 1.015, 1.001)	1.0164, 1.0175, 1)	0.67638, 0.00745, 0.03145, 0.02837)

The distance $$d(_1\hat{\pi },\pi _{\text {sel}}(\hat{\theta })) = 0.0457 $$

### Experimental Data and Results

We now turn our attention to analysis of experimental data from two in vitro datasets, where the raw data are in the form of repeat numbers. For different genes, the repeat numbers, which determine whether the gene is ON or OFF, are different, but this is known and hence phasotypes can be determined from repeat numbers. The estimates/confidence intervals for mutation parameters *p* and *q*, available from Bayliss et al. (2012), relate to mutation rates between repeat numbers, from which mutation rates for phasotypes can again be deduced. For example, if repeat numbers of 8/9 correspond to a certain gene being OFF/ON, then the mutation rate from OFF to ON is simply the mutation from the repeat number 8–9.

From the first data set we have initial (inoculum) and final sample distributions, with an estimated 220 generations between the two. We run our inferential procedure with the prior settings, sample data and inputs detailed in Tables 5 and 6. Note that the priors for the mutation rates for *cj*1437 imply these are much smaller than those for the other two genes; this is because the phasotype switches present in the observed data require a mutation of two tract lengths, so the rates for each mutation of one tract length are multiplied. The other two genes require only one tract length mutation. The vector of estimates is $\hat{\theta }=(_{0}\hat{\dot{\pi }},\hat{n},\hat{p}, \hat{q},\hat{\gamma })$; evaluating model (19) at $\hat{\theta }$ , we obtain the predicted final distribution $\pi _{\text {sel}}(\hat{\theta })$, and we find that $d(_{1}\hat{\pi },\pi _{\text {sel}}(\hat{ \theta }))=0.0656$, which is less than the tolerance $_{1}\epsilon =0.112$ (from (45) with $N=141$). The point estimate of the vector of fitness parameters is $\hat{\gamma }=(1.023,1.008,1.013,1,1.030,1.034,1.022,1.005)$.

**Table 5:** Prior settings for dataset 1

Gene	$\bar{p}_l$ $[p_{l_{*}},p_l^{*}]$ $\times 10^{-4}$	$\bar{q}_l$ $[q_{l_{*}},q_l^{*}]$ $\times 10^{-4}$	$\bar{n}[n_{*},n^{*}]$	$[a_i, b_i]$ for $\varGamma$
*cj*0617	12.30 [9.1, 22.2]	17.88 [11.0, 40.2]	220 [110, 275]	[1, 1.04]
*cj*0685	4.23 [3.0, 5.7]	2.15 [1.4, 2.8]		[1, 1.04]
*cj*1437	$0.0725\, [0.0388,0.2597]$	$0.0045\, [0.0029,0.0107]$		[1, 1.04]
				[1, 1]
				[1.005, 1.06]
				[1.005, 1.06]
				[1, 1.04]
				[1, 1.04]

**Table 6:** Sample data for dataset 1

$_0N$	$_0\epsilon$	$_0\hat{\pi }$	$_1N$	$_1\epsilon$	$_1\hat{\pi }$
300	0.0766	(0.00333, 0.01, 0.00667,	141	0.112	(0.15603, 0.00709, 0.01418,
		0.92333, 0.04333,			0.09220, 0.63121, 0.04255,
		0, 0, 0.01333)			0.04255, 0.01418)

The second dataset is another in vitro dataset, where the conditions of the experiment were the same as the first experiment; hence, it is expected that inferences from the second experiment will reinforce those from the first. However, the time period between initial and final distributions is an estimated 20 generations, as opposed to 220 generations for the first dataset, so this dataset can also be used to answer questions about what happens in the early stages, such as whether most selection happens in the early stages (e.g. fast adaptation to changes in the environment when bacteria are moved from storage to plates). The data and prior settings for this experiment are given in Table 7 where they differ from the previous dataset—the priors for *p* and *q* are the same as before for *cj*0617 and *cj*0685, but for *cj*1437, the relevant switch in the observed data is of only one tract length; hence, the ON–OFF mutations for this gene in this experiment have higher associated rates than in the previous dataset.

**Table 7:** Sample data and prior settings for dataset 2. Also, $_0N = 84$, $_1N=87$, $_0\epsilon = 0.145$, $_1\epsilon = 0.142$

$$\bar{p}_l$$$$[p_{l_{*}},p_l^{*}]$$ ($$\times \, 10^{-4}$$) for *cj*1437	$$\bar{q}_l[q_{l_{*}},q_l^{*}]$$ ($$\times \, 10^{-4}$$) *cj*1437	$$\bar{n}$$ $$[n_{*},n^{*}]$$	$$[a_i, b_i]$$ for $$\varGamma $$	$$_0\hat{\pi }$$	$$_1\hat{\pi }$$
17.88	12.30	20 [10, 25]	[1, 1.6]	(0.0119, 0.0476,	(0.0115, 0.0230,
[11.0, 40.2]	[9.1, 22.2]		[1, 1.6]	0.0000, 0.7738,	0.0230, 0.0690,
			[1, 2]	0.1548, 0.0000,	0.7586, 0.0805,
			[1, 1]	0.0119, 0.0000)	0.0345, 0.0000)
			[1.1, 1.8]		
			[1.05, 2.2]		
			[1, 2.2]		
			[1, 1.6]		

Again, we formed the vector of estimates $\hat{\theta }$ and evaluated the predicted final distribution $\pi _{\text {sel}}(\hat{\theta })$. We find that $d({1}\hat{\pi },\pi _{\text {sel}}(\hat{\theta }))=0.0925$, which is less than the tolerance $_{1}\epsilon =0.142$ (from (45) with $N=87$). As with dataset 1, we conclude that the mutation–selection model is a plausible description of the evolution mechanism for these three genes. For this second dataset, the point estimate of the vector of fitness parameters is $\hat{\gamma }=(1.180,1.172,1.328,1,1.380,1.575,1.354,1.150)$. Notably, the fitness parameters are larger than those of the first dataset, suggesting that selection advantage may be more prominent in the early stages of the experiment. We explore this further in the following section.

### Time Dependence

The estimated fitness parameters for the second dataset (which correspond to a much shorter period of approximately 20 generations) were larger than those obtained from the first dataset. This leads to a hypothesis of biological interest, namely that selection advantage has a larger influence in the initial stages, when the bacteria are adapting to changes in the environment. Thus, the estimates from the first dataset (corresponding to a much longer period of approximately 220 generations) are averaged over a longer period, for most of which the selection advantage is less important. This is a plausible explanation for the lower estimates seen in the first dataset.

To investigate this further, we conducted the following experiment. First, we used the initial distribution from the first dataset as input for the mutation–selection model and ran for 20 generations; for the mutation rates we used the point estimates $\bar{p}$ and $\bar{q}$ as for the first dataset, given in Table 5, and for the fitness parameters we used the point estimates obtained from the second experiment. This provides an interim distribution, $_{0.5}\hat{\pi }$ say. We then apply Algorithm 4.1 using $_{0.5}\hat{\pi }$ as initial distribution and the final distribution taken to be that from the first dataset. The aim is to see if the model can explain this final distribution, and whether the estimates of the fitness parameters are lower (as per our hypothesis). We used the following as inputs for the remaining parameters: the priors for the mutation rates, and the tolerances used, are given in Tables 5 and 6. We chose $\bar{n} =200$ with $[n_{*},n^{*}]=[100,250]$ because 200 is the difference between the expected lengths of the second and first experiments. Initial investigation showed that the mutation-only model could not explain the observed final distribution, and hence there is still evidence of selection advantage over this time period. However, as we expect this advantage to be smaller, we use narrower priors for the selection parameters. Specifically, we used uniform priors over the interval [1, 1.01] for each fitness parameter, which also reflects no preference for a particular phasotype a-priori.

As can be seen from Table 8, the observed and predicted final distributions are within the sampling-variability tolerance. Once again, this shows the ability of our model to explain the observed data and also to provide insight into the switching behaviour and the nature of the selection advantage. Results for the fitness parameters, mutation rates and number of generations are given in Tables 9, 10 and 11, including both point estimates and $95\%$ probability intervals. For example, we see that the posterior probability interval of the number of generations is approximately (210–213), whereas the prior estimate was 200 generations; this also shows the power of using the Bayesian framework to handle uncertainty in such parameters, allowing the model to adapt and provide additional information of interest to biologists beyond point estimates.

**Table 8:** Results for the time-dependence experiment. Here $_1\epsilon = 0.112$

$_1\hat{\pi }$	$\pi _{\text {sel}}(\hat{\theta })$	$d(_1\hat{\pi },\pi _{\text {sel}}(\hat{\theta }))$
(0.15603, 0.00709, 0.01418, 0.09220,	(0.15034, 0.02545, 0.03451, 0.07191,	0.0830
0.63121, 0.04255, 0.04255, 0.01418)	0.59554, 0.05478, 0.04457, 0.02289)

**Table 9:** The minimum, maximum and 95% posterior probability intervals for fitness parameters from time-dependence experiment

$\hat{\gamma }^i$	$\min \gamma ^i$	$\max \gamma ^i$	95% posterior probability intervals for $\gamma ^i$
1.004021	1	1.00998	[1, 1.00925]
1.001056	1	1.00869	[1, 1.00618]
1.000296	1	1.00586	[1, 1.00410]
1.006620	1.00228	1.00994	[1.00425, 1.00961]
1.007894	1.00610	1.00999	[1.00708, 1.00982]
1.000000	1	1.00552	[1, 1.00341]
1.002977	1	1.00986	[1, 1.00895]
1.002558	1	1.00941	[1, 1.008791]

**Table 10:** The minimum, maximum and 95% posterior probability intervals ($\times \, 10^{-4}$) for mutation rates from time-dependence experiment

Gene	$\hat{p}_l$	$\min p_l$	$\max p_l$	95% interval ($p_l$)	$\hat{ q}_l$	$\min q_l$	$\max q_l$	95% interval ($q_l$)
*cj*0617	12.308	9.135	17.580	9.534, 15.762	16.257	11.084	25.958	[11.727, 21.948]
*cj*0685	4.126	3.002	5.619	3.112, 5.248	2.152	1.405	2.800	[1.580, 2.723]
*cj*1437	0.0724	0.0389	0.127	0.0423, 0.109	0.00453	0.00294	0.00775	[0.00310, 0.00627]

**Table 11:** The minimum, maximum and 95% posterior probability interval for the number of generations from the time-dependence experiment

$\hat{n}$	$\min g_{\tilde{\eta }}(n)$	$\max g_{\tilde{\eta }}(n)$	2.5/97.5 percentiles from $g_{\tilde{\eta }}(n)$
212	145	250	168, 246

## Discussion and Conclusions

In this work, we consider two models (mutation and mutation–selection) for describing time evolution of a bacteria population. The models are accompanied by algorithms for determining whether they can explain experimental data and for estimating unobservable parameters such as fitness. In the case of the mutation–selection model, we propose an algorithm inspired by Approximate Bayesian Computation (ABC) to link the model and data. The approach considered gives microbiologists a tool for enhancing their understanding of the dominant mechanisms affecting bacterial evolution which can be used, e.g. for creating vaccines. Here, we limit ourselves to illustrative examples using in vitro data for phase variable (PV) genes of *C. jejuni* aimed at demonstrating how the methodology works in practice; more in depth study of PV genes will be published elsewhere. We note that the models together with the methodology linking the models and the data can be applied to other population dynamics problems related to bacteria. In particular, it is straightforward to adjust the methodology presented if considering repeat numbers instead of phasotypes.

The calibration of the models is split into two steps. First, the very efficient algorithm from Sect. 3 is applied to verify whether data for particular genes can be explained by the mutation model. This allows us to reduce the number of genes to which the mutation–selection model should be applied. The second step is calibration of the mutation–selection model for the remaining genes using the ABC-type algorithm from Sect. 4. In both steps, we take into account experimental errors and sample sizes. We note that, due to its computational complexity, the ABC algorithm is realistic to apply in the case of relatively small number of genes (2–6). We also note that if one wants to model simultaneously a large number of genes with dependent behaviour (e.g. if one needs to simultaneously model all 28 PV genes of *C. jejuni* strain NCTC11168, where the state space is of order $10^{17}$) then a space-continuous model should be used instead of discrete-space models considered here. Development of such space-continuous models together with calibration procedures for them is a possible topic for future research.

Further development of the presented approach can include enhancing the models by adding a description of bottlenecks and, consequently, proposing algorithms to answer questions about the presence of bottlenecks during bacterial evolution. It is also of interest to consider continuous-time counterparts of the discrete-time models studied here and thus take into account random bacterial division times. (For this purpose, e.g. ideas from Caravagna et al. (2013) and D’Onofrio (2013) can be exploited.) It will lead to models written as differential equations for which the discrete models of this paper are approximations.

The proposed ABC algorithm for estimating fitness parameters can be further developed in a number of directions. For instance, the computational costs of this algorithm grow quickly with an increase in the number of genes, and recent improvements to ABC, such as adaptive methods based on importance sampling using sequential Monte Carlo (e.g. Beaumont et al. 2009; del Moral et al. 2012) could potentially be exploited to make the algorithm more efficient. We also left for future work analysis of convergence of the considered ABC-type algorithm.

One of the assumptions we used is that mutations of individual genes happen independently of each other [see (11)] and that mutation rates do not change with time/environment, which are commonly accepted hypotheses in microbiology. At the same time, it is interesting to test the environmentally directed mutation hypothesis (see Lenski et al. 1989 and references therein), i.e. to verify whether upon relaxing assumptions on the transitional probabilities the mutation model can explain the data for the three genes considered in our experiments of Sect. 5. It is clear from our study (see also Bayliss et al. 2012) that under assumption (11) the mutation model cannot explain the data. Herein, we then test whether these three genes can be explained by a combination of mutation and selection. However, it is formally possible that the observed patterns could be explained by allowing for dependence of mutations. The data assimilation approach of Sect. 4 can be modified to test for dependence of mutations.

Though the main objective of the paper was to propose tractable models for bacterial population evolution together with their robust calibration, a number of biologically interesting observations were made. First, we saw in Sect. 3.2 that in the considered in vitro experiment some of the PV genes can be explained by the mutation model and some are not and hence were subject of further examination via the mutation–selection model. A plausible explanation, and indeed an expected outcome, is that genes vary in their responses to selection with the mutation-only genes not contributing to bacterial adaptation in this particular experimental set up. In Sect. 5, we studied three genes which did not pass the test of Sect. 3. We started by verifying whether the data can be explained by the mutation–selection model with fitness parameters being assigned to the individual genes (Assumption 2.3) rather than to specific phasotypes. (Note that three genes can generate eight phasotypes; 111, 110, 100, etc.) This hypothesis was rejected implying an important biological consequence namely that selection acts on phasotypes and there is a dependence between the three genes, i.e. adaptivity to a new environment in this case relies on a particular, coordinated configuration of states of the three genes. Next (Sect. 5.3) we estimated fitness parameters of the mutation–selection model (without imposing Assumption 2.3) and thus showed that the data can be explained by this model, i.e. these genes’ behaviour can be described using a combination of the selection and mutation mechanisms but not mutations alone. The treatment encompassed by the in vitro experiment had only one change of environment when bacteria were moved from a storage environment to sequential replication on plates. It was then natural to expect that adaptation happens soon after bacteria are placed on plates resulting in a requirement for rapid adaptation to this major environmental shift whereas sequential replication on plates maintains a constant selective regime. Using the mutation–selection model with time-dependent fitness coefficients, in Sect. 5.4 we confirmed this hypothesis using data at an intermediate time point. This is a remarkable demonstration of the usefulness of the approach proposed in this paper.

## A Proof of Proposition 2.4

We first prove the following lemma which gives the stationary distribution for the mutation model (19) for a single gene.

### Lemma A.1

Let Assumption 2.2 hold. Then, the unique stationary distributions $^{\infty }\pi _{\text {sel,}i}$ for single genes *i* individually described by (19) are equal to

46 $$\begin{aligned} ^{\infty }\pi _{\text {sel,}i}^{1}= & {} \dfrac{2\gamma _{i}^{1}q_{i}}{ (1-q_{i})\varDelta \gamma _{i}+\gamma _{i}^{1}(p_{i}+q_{i})+\sqrt{(\gamma _{i}^{1}p_{i}+\gamma _{i}^{2}q_{i})^{2}+2(\gamma _{i}^{1}p_{i}-\gamma _{i}^{2}q_{i})\varDelta \gamma _{i}+\left( \varDelta \gamma _{i}\right) ^{2}}}, \,\,\,\,\,\,\,\,\,\,\, \nonumber \\ ^{\infty }\pi _{\text {sel,}i}^{2}= & {} 1-\ ^{\infty }\pi _{\text {sel,}i}^{1}\ ,\ \varDelta \gamma _{i}=\gamma _{i}^{2}-\gamma _{i}^{1}\ . \end{aligned}$$

### Proof

It follows from (19) with $\ell =1$ that the first component of the stationary distribution for an *i*th gene $^{\infty }\pi _{\text {sel,}i}^{1}$ of $^{\infty }\pi _{\text {sel,} i}=(^{\infty }\pi _{\text {sel,}i}^{1},^{\infty }\pi _{\text {sel,}i}^{2})$ should satisfy the following quadratic equation

47 $$\begin{aligned} (p_{i}+q_{i}-1)\varDelta \gamma _{i}\left[ ^{\infty }\pi _{\text {sel,}i}^{1} \right] ^{2}+\left[ (1-q_{i})\varDelta \gamma _{i}+\gamma _{i}^{1}(p_{i}+q_{i}) \right] \ ^{\infty }\pi _{\text {sel,}i}^{1}-\gamma _{i}^{1}q_{i}=0. \end{aligned}$$

By simple algebra, it is not difficult to establish that under Assumption 2.2 (also recall that all $\gamma _{i}^{j}>0)$ Eq. (47) always has only one solution which is between 0 and 1 and that it is equal to the expression from (46). Lemma A.1 is proved. $\square$

### Proof of Proposition 2.4

We need to check that $^{\infty }\pi _{\text {sel}}$ from (30) satisfies the equation for the stationary distribution

48 $$\begin{aligned} ^{\infty }\pi _{\text {sel}}=\frac{^{\infty }\pi _{\text {sel}}TI_{\gamma }}{ \gamma \cdot \ ^{\infty }\pi _{\text {sel}}T} \end{aligned}$$

or equivalently

49 $$\begin{aligned} ^{\infty }\pi _{\text {sel}}\left[ ^{\infty }\pi _{\text {sel}}T\gamma ^{\top } \right] -\ ^{\infty }\pi _{\text {sel}}TI_{\gamma }=0, \end{aligned}$$

which we prove by induction. By Lemma A.1, (49) is true for $\ell =1.$ Assume that (49) is true for all $\ell \le k.$ Consider $\ell =k+1.$ Using (25), (26), and (11 ), we obtain

$$\begin{aligned}&^{\infty }\pi _{\text {sel}}[^{\infty }\pi _{\text {sel}}T\gamma ^{\top }]-\ ^{\infty }\pi _{\text {sel}}TI_{\gamma } \\&\quad =(\ ^{\infty }\pi _{\text {sel,}1}\otimes \cdots \otimes \ ^{\infty }\pi _{ \text {sel,}k+1}) \\&\qquad [(\ ^{\infty }\pi _{\text {sel,}1}\otimes \cdots \otimes \ ^{\infty }\pi _{\text {sel,}k+1})(T_{1}\otimes \cdots \otimes T_{k+1})(\gamma _{1}\otimes \cdots \otimes \gamma _{k+1})^{\top }] \\&\qquad -(\ ^{\infty }\pi _{\text {sel,}1}\otimes \cdots \otimes \ ^{\infty }\pi _{ \text {sel,}k+1})(T_{1}\otimes \cdots \otimes T_{k+1})(I_{\gamma _{1}}\otimes \cdots \otimes I_{\gamma _{k+1}}). \end{aligned}$$

$$\square $$

By the mixed-product and bilinear properties of the Kronecker product, we get

$$\begin{aligned}&^{\infty }\pi _{\text {sel}}[^{\infty }\pi _{\text {sel}}T\gamma ^{\top }]-\ ^{\infty }\pi _{\text {sel}}TI_{\gamma } \\&\quad =\ ^{\infty }\pi _{\text {sel,}1}[\ ^{\infty }\pi _{\text {sel,} 1}T_{1}(\gamma _{1})^{\top }]\otimes \cdots \otimes \ ^{\infty }\pi _{\text { sel,}k+1}[\ ^{\infty }\pi _{\text {sel,}k+1}T_{k+1}(\gamma _{k+1})^{\top }] \\&\qquad -\ ^{\infty }\pi _{\text {sel,}1}T_{1}I_{\gamma _{1}}\otimes \cdots \otimes \ ^{\infty }\pi _{\text {sel,}k+1}T_{k+1}I_{\gamma _{k+1}} \\&\quad =(\ ^{\infty }\pi _{\text {sel,}1}[\ ^{\infty }\pi _{\text {sel,} 1}T_{1}(\gamma _{1})^{\top }]\otimes \cdots \otimes \ ^{\infty }\pi _{\text { sel,}k}[\ ^{\infty }\pi _{\text {sel,}k}T_{k}(\gamma _{k})^{\top }] \\&\qquad -\ ^{\infty }\pi _{\text {sel,}1}T_{1}I_{\gamma _{1}}\otimes \cdots \otimes \pi _{k}T_{k}I_{\gamma _{k}})\otimes \ ^{\infty }\pi _{\text {sel,}k+1}[\ ^{\infty }\pi _{\text {sel,}k+1}T_{k+1}(\gamma _{k+1})^{\top }] \\&\qquad +\ ^{\infty }\pi _{\text {sel,}1}T_{1}I_{\gamma _{1}}\otimes \cdots \otimes \ ^{\infty }\pi _{\text {sel,}k}T_{k}I_{\gamma _{k}} \\&\qquad \otimes (^{\infty }\pi _{\text {sel,}k+1}[^{\infty }\pi _{\text {sel,} k+1}T_{k+1}(\gamma _{k+1})^{\top }]-^{\infty }\pi _{\text {sel,} k+1}T_{k+1}I_{\gamma _{k+1}}), \end{aligned}$$

where the difference in the first term on the right-hand side is zero because of the induction assumption and the difference in the second term is zero due to Lemma A.1. Hence, $^{\infty }\pi _{\text {sel}}$ from (30) satisfies (48) for $\ell =k+1,$ and therefore (30) is proved for any $\ell .$ It is also not difficult to check that $\sum _{i=1}^{2^{\ell }}\pi _{\text {sel}}^{i}=1.$ Proposition 2.4 is proved.
